# Prucalopride, a serotonin type 4 receptor agonist, induces fast anxiolytic/antidepressant effects and concomitant changes in the gut microbiota

**DOI:** 10.1038/s41522-026-00928-6

**Published:** 2026-02-04

**Authors:** Sofia Cussotto, Salma R. Abdennebi, Isabelle Etting, Christine A. Denny, René Hen, Romain Colle, Emmanuelle Corruble, Jean-Claude Alvarez, Denis J. David, Indira Mendez-David

**Affiliations:** 1 Université Paris-Saclay, UVSQ, Centre de recherche en Epidémiologie et Santé des Populations (CESP), UMR 1018, CESP-Inserm, Team Moods, Faculté de Pharmacie, Bâtiment Henri Moissan, Orsay, France; 2 Department of Pharmacology and Toxicology, Raymond Poincaré Hospital, GHU AP-HP, UVSQ/Paris-Saclay, Garches, France; 3 Inserm U-1018, CESP, Team MOODS, Paris-Saclay/Versailles University, Garches, France; 4 Department of Psychiatry, Columbia University Irving Medical Center, New York; Division of Systems Neuroscience, New York State Psychiatric Institute, New York, NY USA; 5 Université Paris-Saclay, Centre de recherche en Epidémiologie et Santé des Populations (CESP), MOODS UMR1018, CESP-Inserm, Team Moods, Faculté de Médecine, Le Kremin-Bicêtre, France; 6 Service Hospitalo-Universitaire de Psychiatrie, Hôpital de Bicêtre, Hôpitaux Universitaires Paris-Saclay, Assistance Publique-Hôpitaux de Paris, Le Kremlin-Bicêtre, France

**Keywords:** Microbiota, Microbiome

## Abstract

Major Depressive Disorder (MDD) affects around 20% of people globally and is often comorbid with anxiety. This study investigates prucalopride, a serotonin type 4 receptor (5-HT_4_R) agonist approved for constipation, as a fast-acting anxiolytic/antidepressant using a mouse model of stress, based on corticosterone (CORT) administration. Behavioral effects of prucalopride (0.5 and 1.5 mg/kg/day) were compared to fluoxetine, a common SSRI, over 7 (subchronic) and 28 (chronic) days. Prucalopride showed faster and more significant improvements in emotionality scores than fluoxetine, reversing CORT-induced behavioral changes within 7 days. Gut microbiota analysis revealed CORT-induced changes at the subchronic timepoint. While chronic prucalopride did not alter microbial alpha diversity, it significantly shifted microbial composition (beta-diversity). Notably, prucalopride restored levels of the genus *Ruminococcus*, which were depleted by CORT. Our findings highlight prucalopride’s rapid anxiolytic and antidepressant-like effects and its impact on gut microbiota, supporting the potential of 5-HT_4_R-targeting molecules as therapeutic options for psychiatric disorders.

## Introduction

Major Depressive Disorder (MDD) is one of the most common mental health disorders, affecting approximately 20% of individuals worldwide at some point in their lives^1^. During Major Depressive Episodes (MDE), about 2/3 of patients experience significant levels of anxiety^1^. Current treatment options for MDE, especially when comorbid with anxiety, face several challenges. Monoaminergic antidepressants for instance, show a delayed onset of action leaving patients vulnerable during the critical early weeks of treatment^2^. Benzodiazepines, rapid-acting GABAergic anxiolytics, primarily address anxiety but provide minimal relief for depressive symptoms^3^. Furthermore, their use is associated with significant adverse effects, including sedation, cognitive impairment, and a high risk of dependence^4^. Therefore, there is a pressing need for the innovation and development of new treatments that are both fast-acting and safe, representing a crucial step forward for public health.

Recent studies have identified the serotonin type 4 receptor (5-HT_4_R) as a promising target for treating depression and anxiety^5–7^. This metabotropic receptor is primarily expressed in the limbic system of both primates and rodents, including the hypothalamus, hippocampus, nucleus accumbens, the amygdala and the frontal cortex, consistent with its role in emotions regulation [for review^5^]. In clinical settings, a 5-HT_4_R genetic polymorphism has been associated with reduced response to conventional antidepressants^8^. Additionally, positron emission tomography scanning has highlighted a reduced expression of 5-HT_4_R in individuals diagnosed with MDD compared to healthy controls^9^. Overall, these findings emphasize the critical role of 5-HT_4_R in the pathophysiology of MDD and provide the fundamental bases to support ongoing research into 5-HT_4_R agonists as a promising direction for developing more effective treatments in the context of mood disorders. In this context, we have recently shown that a 5-HT_4_R agonist, RS67333, exhibits rapid anxiolytic and antidepressant effects in mouse models^5–7^. Nonetheless, studying these effects in humans has been constrained by the adverse side effects. Interestingly, prucalopride, a selective and a high-affinity 5-HT_4_R partial agonist (pKi=8.6^10^), with good brain penetration^11^, is approved by both the *Food and Drug Administration* and the *European Medicines Agency* for the treatment of chronic idiopathic constipation (CIC)^10^. A recent study utilizing anonymized data from US electronic health records found that treatment with prucalopride was associated with significantly lower incidence of depression over the following year compared to two alternative anti-constipation medications^12^. In rodents, while a previous study demonstrated prucalopride’s prophylactics effect against stress^13^ and increases firing of serotonin (5-HT) neurons, a mechanism likely contributing to its antidepressant activity, its efficacy in a mouse model of anxiety/depression has not yet been implemented^14^.

In addition to its expression in the brain, 5-HT_4_Rs are highly abundant in the gastrointestinal tract, where they play a key role in regulating gut motility, secretion, and sensory functions^15,16^. As increasing evidence is highlighting a role for the microbiota-gut-brain axis in psychiatric disorders^17,18^, the question arises as to whether prucalopride might exert potential antidepressant/anxiolytic effects via the gut-brain axis. No studies to date have explored the effects of prucalopride on microbiota composition.

To address this gap in knowledge, the present study aimed to characterize the effects of prucalopride in the chronic corticosterone (CORT) stress mouse model, focusing on both central and peripheral outcomes. The central readout involved behavioral screening of emotion-related responses, while the peripheral investigation centered on the gut microbiota using 16S sequencing. This technique targets the 16S ribosomal RNA (rRNA) gene, which is highly conserved and present in all bacteria, making it ideal for identifying and characterizing each microorganism^19^. Observations along the gut-brain axis were conducted at two distinct timepoints, allowing a comparison of subchronic *versus* chronic effects. Overall, this research has the potential to provide novel insights, paving the way for future clinical investigations into the therapeutic role of 5-HT_4_R-targeting molecules for the treatment of MDD.

## Results

### Corticosterone administration increases plasma corticosterone levels and impacts the gut microbiota composition

**Fig. 1:**
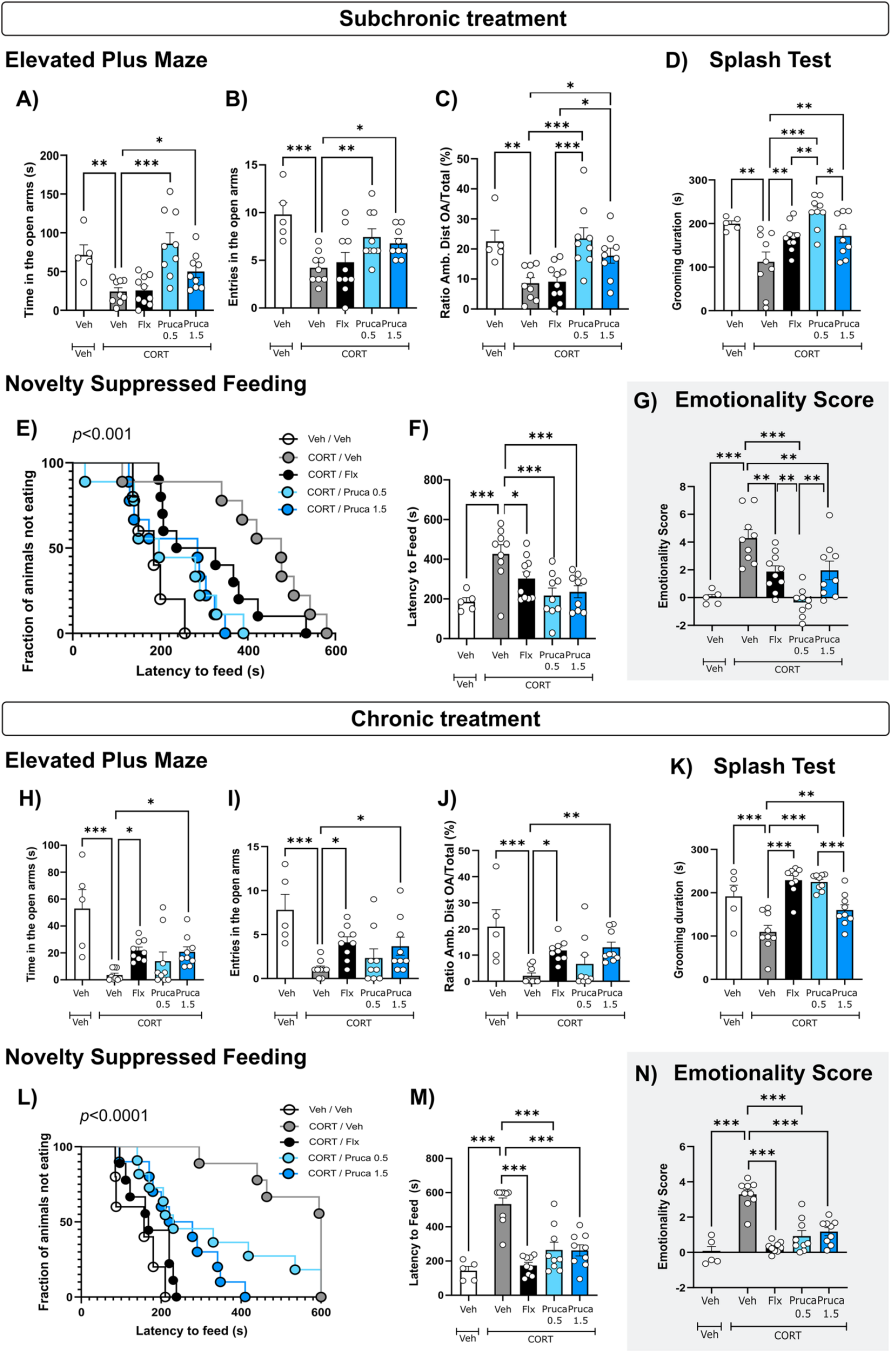
Effects of a subchronic or chronic prucalopride treatment on corticosterone-induced anxiety- and depression-related behaviors. Effects of a subchronic (**A**–**G**) or chronic (**H**–**N**) prucalopride (Pruca_0.5_, Pruca_1.5_) or fluoxetine (Flx) administration on corticosterone (CORT) -induced anxiety-like behaviors in the Elevated Plus Maze (EPM). Anxiety is expressed as mean time spent in seconds (**A** or **H**) or number of entries (**B** or **I**) in the open arms or as mean ratio of ambulatory distance in the center/total ambulatory distance (**C** or **J**). Effect of a subchronic (**D**) or chronic (**K**) prucalopride (Pruca_0.5_, Pruca_1.5_) or fluoxetine (Flx) administration on corticosterone (CORT)-induced depression-related behaviors in the Splash Test (ST). Depression-related behavior is expressed as mean duration of grooming in seconds (**D** or **K**). Effects of a subchronic (**E**, **F**) or chronic (**L**, **M**) prucalopride (Pruca_0.5_, Pruca_1.5_) or fluoxetine (Flx) administration on corticosterone (CORT) -induced anxiety and depression-like behaviors in the Novelty Suppressed Feeding (NSF) paradigm. Results are cumulative survival of animals that have not eaten over 10 min (**E** or **L**) or mean of latency to feed in seconds (**F** or **M**). Effects of a subchronic (**G**) or chronic (**N**) prucalopride (Pruca_0.5_, Pruca_1.5_) or fluoxetine (Flx) administration on corticosterone (CORT)-induced anxiety/depression-like phenotype. Test Z-values (Elevated Plus maze, Splash test and Novelty Suppressed Feeding) are then calculated by averaging individual Z-scores and averaged to obtain emotionality Z-score. Data are expressed as mean ± SEM (Veh/Veh *n* = 5, CORT/Veh *n* = 9, CORT/Flx *n* = 9–10, CORT/Pruca_0.5_ *n* = 9, CORT/Pruca_1.5_ *n* = 9). *p < 0.05; **p < 0.01; ***p < 0.001 versus appropriate group.

To confirm the biological validity of our model, we measured circulating CORT levels following 5 and 8 weeks of CORT administration in the drinking water. Consistent with previous findings^20^, the CORT/Veh group showed an increase in CORT levels after 5 and 8 weeks of treatment compared to Veh-treated group. This increase was reversed in all other treatment groups (CORT/Flx, CORT/Pruca_0.5_, CORT/Pruca_1.5_; Fig. S2). As previously described, CORT treatment increased anxiety- and depressive-like behavior as observed with the increase in emotionality score after 5 and 8 weeks of treatment^7,20,21^. Indeed, at both time points, in the EPM, CORT decreased the time spent and the number of entries in the open arms the ratio of ambulatory distance in the open arms divided by total distance (Fig. 1A, B). Moreover, chronic CORT decreased the grooming duration in the ST (Fig. 1D) and increased latency to feed in the NSF (Fig. 1E, F).

While most research to date has focused on the microbiota’s ability to regulate CORT levels^22,23^, the impact of chronic CORT supplementation on the gut microbiota itself remains underexplored. To this end, we compared specifically the gut microbiota of Veh-treated and CORT-treated mice. CORT administration induced distinct, time-dependent alterations in the gut microbiota, as detailed in Fig. S3. Specifically, the Chao1 index (a species richness estimator that considers both the observed species and the contribution of rare species in the community) for alpha-diversity was significantly decreased by CORT after 5 weeks but not 8 weeks of treatment. Also, CORT-receiving mice clustered apart from Veh mice on the beta-diversity Bray-Curtis matrix, independent of the timepoint. The genera *Clostridia UCG014* and *Ruminococcus* were significantly depleted by CORT supplementation at both timepoints.

### The 5-HT_4_R agonist prucalopride induces differential dose-related anxiolytic/antidepressant effects following both subchronic and chronic treatments

To assess the potential fast anxiolytic/antidepressant effect of prucalopride, a battery of behavioral tests was conducted after 3 days of subchronic treatment. In the EPM, prucalopride at both doses significantly increased the time spent and the number of entries in the open arms, highlighting an anxiolytic-like effect, which was not observed for fluoxetine (Fig. 1A, B). The ratio of ambulatory distance in the open arms divided by total distance was increased by both doses of prucalopride, indicating an anxiolytic-like effect (Fig. 1C). The ST revealed a significant increase in grooming duration for both doses of prucalopride, an effect also observed in the fluoxetine group (Fig. 1D). Subsequently, we evaluated these mice in the NSF test to measure hyponeophagia, an index of anxiolytic/antidepressant-like activity. Prucalopride, at both tested doses, significantly decreased the latency to feed (Fig. 1E, F) without affecting the home-cage food consumption (Fig. S4). The NSF effect seemed independent of bodyweight changes across groups, which were not significant (Fig. S5), suggesting no changes in feeding behavior. Overall, prucalopride at both doses normalized the emotionality score increase induced by chronic CORT after a week of treatment (Fig. 1G). Notably, at the lowest dose tested, prucalopride elicited a more pronounced anxiolytic/antidepressant-like effect.

The same behavioral tests were administered following chronic treatment. In the EPM, prucalopride (1.5 but not 0.5 mg/kg) exhibited an anxiolytic effect (Fig. 1H–J). This effect was evident across several measures, including time spent in the open arms, entries into the open arms, and the ratio of ambulatory distance in the open arms to total distance. These results were comparable to those observed with fluoxetine. The ST revealed a dose-dependent significant increase in grooming duration for prucalopride as compared to Veh/CORT mice (Fig. 1K). In the NSF, both doses of prucalopride reduce the latency time to reach the food pellet (Fig. 1L, M) without affecting home-cage food consumption (Fig. S4). Overall, the anxiolytic/antidepressant-like effects observed after a week of treatment with prucalopride was sustained following chronic administration, as evidenced by the normalization of the emotionality score at both tested doses (Fig. 1N). Additionally, we confirmed that chronic treatment with fluoxetine reversed chronic CORT-induced increase in emotionality score^23^.

### Following a subchronic treatment gut microbiota shifts are not prucalopride-dependent but likely linked to the depressive- and anxious-like phenotype (CORT model)

**Fig. 2:**
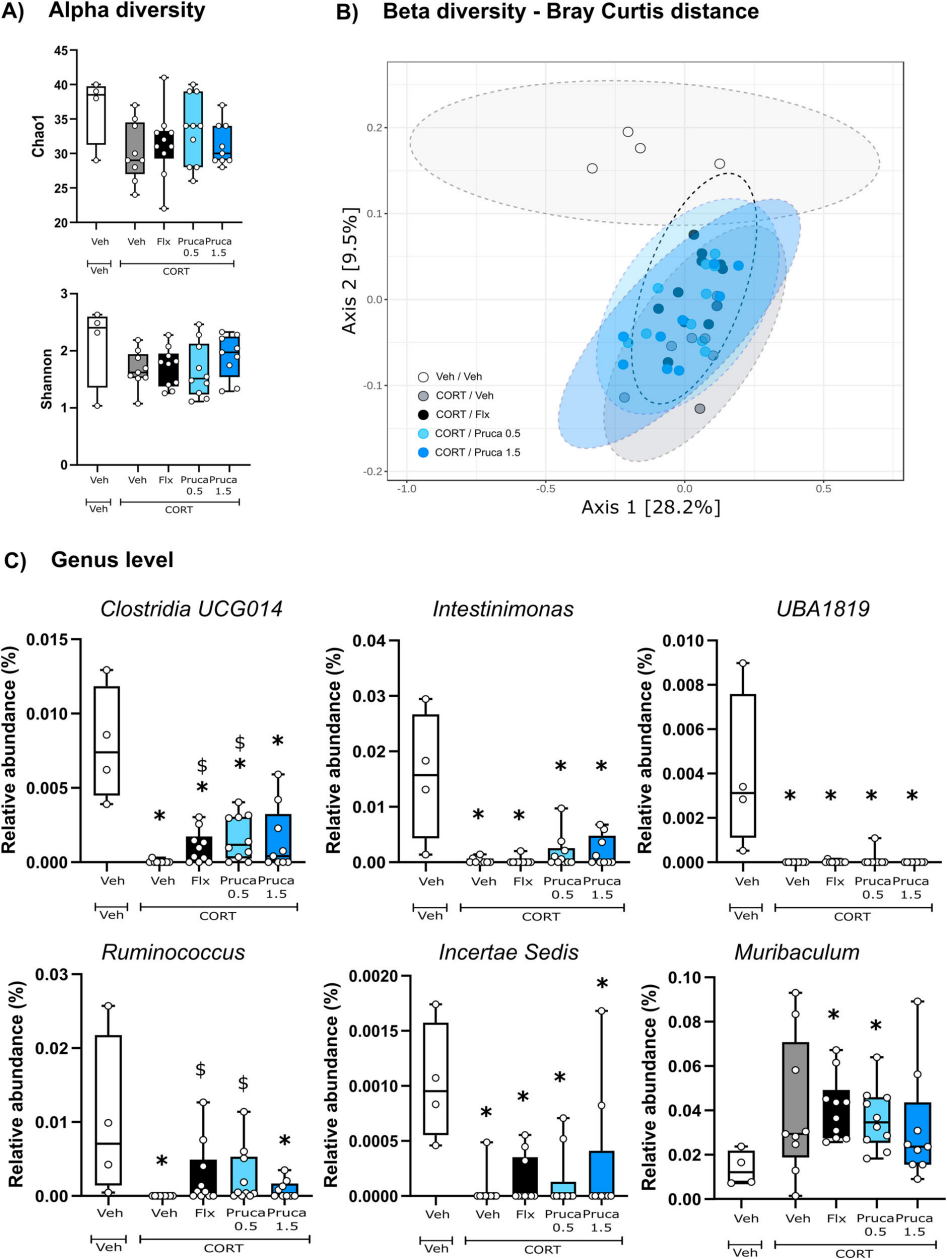
Gut microbiota changes are likely due to CORT treatment rather than prucalopride following a subchronic administration of the 5-HT_4_R agonist. **A** Alpha-diversity: Kruskal-Wallis test for Chao1 and Shannon not significant. **B** Beta-diversity, principal coordinate analysis of Bray-Curtis compiled distance matrix of all genera. **C** Microbiota changes at genus level. *p < 0.05 vs Veh/Veh and ^$^*p* < 0.05 vs CORT/Veh. Data are expressed as median + min-to-max values (Veh/Veh *n* = 4, CORT/Veh *n* = 9, CORT/Flx *n* = 10, CORT/Pruca_0.5_ *n* = 10, CORT/Pruca_1.5_ *n* = 9). Data is analyzed using Kruskal-Wallis non-parametric test followed by Mann–Whitney U-test and corrected for multiple comparisons using the Benjamin-Hochberg false discovery rate (FDR) method.

Two distinct timepoints, corresponding to behavioral assessments, were selected for microbiota compositional analysis. First, the gut microbiota was characterized after 1 week of fluoxetine or prucalopride treatment. At this timepoint, genus level analysis revealed no changes in alpha-diversity metrics (Fig. 2A), which reflects the diversity within each group. Specifically, neither Chao1, which focuses on the number of species, nor Shannon, which accounts for both species’ richness and evenness, were affected. However, beta-diversity analysis, which refers to the comparison of microbial composition between different environments, indicated that the microbiota composition in CORT-treated mice, regardless of fluoxetine or prucalopride administration, was significantly different from that of Veh/Veh controls (Fig. 2B). At the individual genus level, we observed a significant reduction in *Clostridia UCG014*, *Intestinimonas*, *UBA1819*, and *Incertae Sedis* in all CORT-treated groups compared to the Veh-treated group. Additionally, *Muribaculum* was specifically increased in the CORT/Flx and CORT/Pruca_0.5_ relative to Veh/Veh mice. *Ruminococcus* was depleted in CORT/Veh and CORT/Pruca_1.5_ groups as compared to Veh/Veh and was restored in the CORT/Flx and CORT/Pruca_0.5_ groups relative to the CORT/Veh group (Fig. 2C).

### Following a chronic administration the genus *Ruminococcus* is restored in prucalopride-receiving mice

**Fig. 3:**
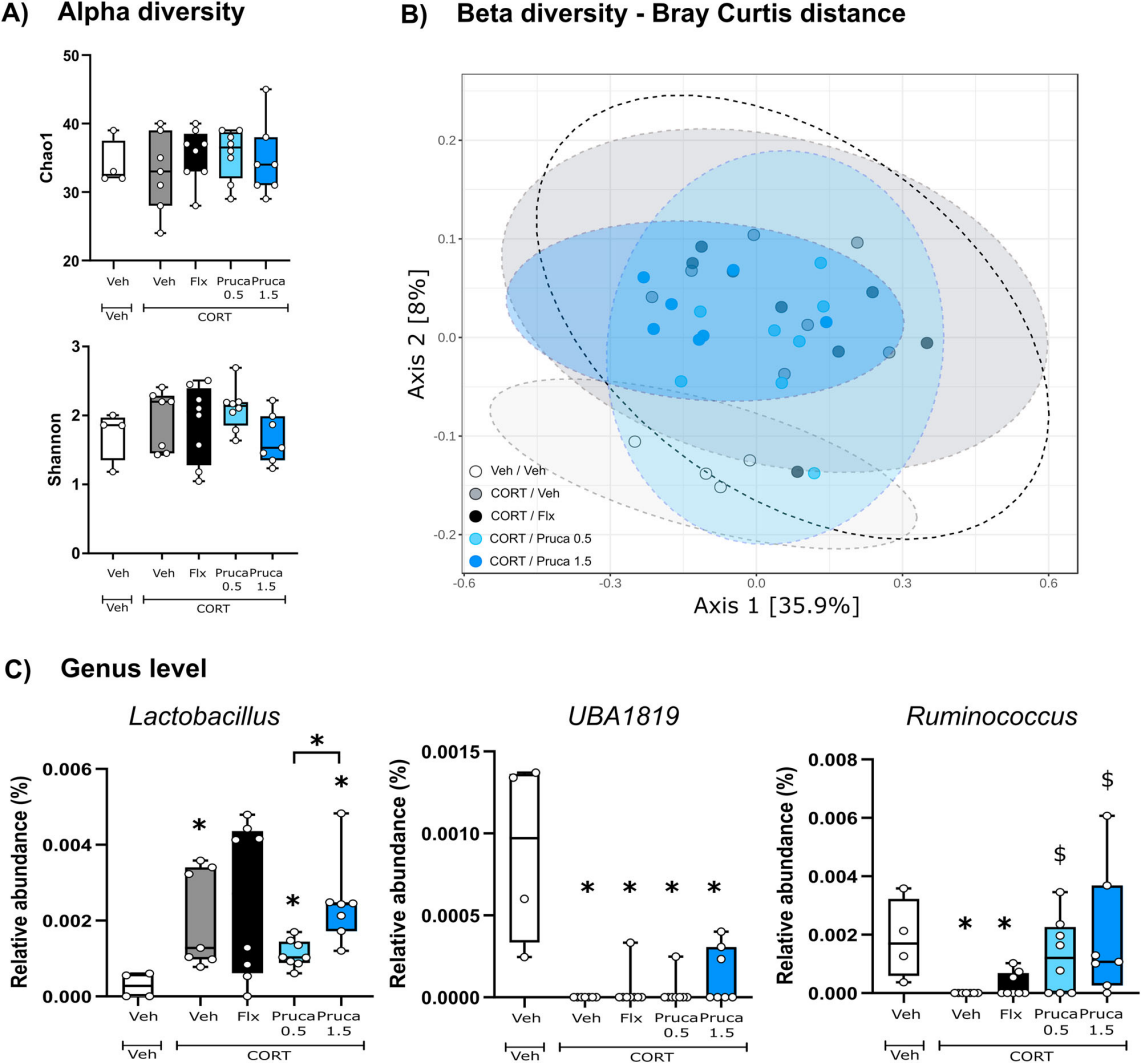
Gut microbiota changes following a chronic administration of the 5-HT_4_R agonist prucalopride. **A** Alpha-diversity: Kruskal-Wallis test for Chao1 and Shannon not significant. **B** Beta-diversity, principal coordinate analysis of Bray-Curtis compiled distance matrix of all genera. **C** Microbiota changes at genus level. *p < 0.05 vs Veh/Veh and ^$^*p* < 0.05 vs CORT/Veh. Data are expressed as median + min-to-max values (Veh/Veh *n* = 4, CORT/Veh *n* = 7, CORT/Flx *n* = 8, CORT/Pruca_0.5_ *n* = 8, CORT/Pruca_1.5_ *n* = 7). Data is analyzed using Kruskal-Wallis non-parametric test followed by Mann–Whitney U-test and corrected for multiple comparisons using the Benjamin-Hochberg false discovery rate (FDR) method.

After 28 days of fluoxetine or prucalopride treatment, microbiota composition was re-analyzed. Consistent with the findings from the subchronic timepoint, no changes in alpha-diversity metrics were detected (Fig. 3A). However, beta-diversity analysis using the Bray-Curtis index revealed the same CORT-related effect as noted at earlier timepoint (compared to Veh/Veh), along with distinct separation between the CORT/Flx and CORT/Pruca_1.5_ groups, as well as between the CORT/Pruca_0.5_ and CORT/Pruca_1.5_ groups (Fig. 3B). At the genus level, a significant reduction in *UBA1819* was observed across all CORT-treated groups compared to Veh-treated group. *Lactobacillus* levels were specifically increased in the CORT/Veh, CORT/Pruca_0.5_, and CORT/Pruca_1.5_ groups relative to Veh/Veh mice. The same genus was also increased in the CORT/Pruca_1.5_ compared to the CORT/Pruca_0.5_ mice. *Ruminococcus* remained depleted in the CORT/Veh group but was restored following chronic prucalopride treatment at both doses, an effect not observed with fluoxetine (Fig. 3C). These data suggest a prucalopride-dependent effect in restoring *Ruminococcus* levels.

## Discussion

Here, using a mouse stress model, we sought to evaluate prucalopride, a clinically approved 5-HT_4_R agonist, as a rapid anxiolytic/antidepressant and to assess its effects on the gut microbiota. These data indicate that prucalopride produced rapid and robust anxiolytic- and antidepressant-like effects compared to fluoxetine, normalizing CORT-induced emotional alterations within a week. In parallel, CORT exposure impacted gut microbiota composition, and prucalopride selectively modulated beta-diversity without affecting alpha-diversity. Notably, prucalopride consistently restored Ruminococcus levels, highlighting this genus as a potential mediator of the treatment’s gut–brain axis effects.

Current antidepressant treatments face a high rate of non-responsiveness among patients^24^, a delayed onset of therapeutic effects, and a range of undesirable side effects^25^ underscoring the critical need for the development of novel antidepressants^26^. Although benzodiazepines, which enhance γ-aminobutyric acid (GABA) neurotransmission^27,28^, have proven effective in the treatment of most anxiety disorders, their long-term daily use is associated with side effect. Consequently, they are often replaced by monoaminergic antidepressants such as selective serotonin reuptake inhibitors (SSRIs), highlighting the central role of serotonin not only in depression but also in anxiety^27,28^. Emerging evidence^6,7,29–31^, along with the present findings, confirm that 5-HT_4_R agonists act as rapid anxiolytics and antidepressants. Our previous research demonstrated that RS-67,333, a 5-HT_4_R partial agonist, could induce rapid anxiolytic effects comparable to those of diazepam^6^ and achieve faster antidepressant-like effects than fluoxetine^7^. However, while the potential for investigating RS-67,333 in humans is constrained by its associated adverse side effects, prucalopride, a selective high-affinity partial agonist of the 5-HT_4_R with excellent brain penetration^11^, offers a promising alternative. As previously mentioned, prucalopride is currently approved for the treatment of CIC^10^ and a recent study based on electronic health records showed prucalopride’s association with reduced likelihood of experiencing a first depressive episode^12^. In our study, while a subchronic treatment with fluoxetine or prucalopride induced anxiolytic/antidepressant-like activity in the NSF and ST, only the 5-HT_4_R agonist resulted in anxiolytic-like activity in the EPM. Subchronic treatment with prucalopride effectively reversed the CORT-induced increase in emotionality after just one week of administration at both tested doses. Notably, the anxiolytic and antidepressant-like effects were more pronounced at the lower dose compared to those observed in fluoxetine-treated animals. A longer duration of treatment (28 days) was required for fluoxetine to exert anxiolytic-like effects comparable to 7 days of prucalopride treatment. Our study further confirmed that the anxiolytic and antidepressant-like effects observed following chronic 5-HT_4_R activation are comparable to those achieved with fluoxetine. To our knowledge, the present study is the first to clearly demonstrate fast anxiolytic-like activity of prucalopride in a mouse model of stress and provide novel insights on the potential repurposing of prucalopride for anxiety and depressive disorders.

Considering that prucalopride is used for the treatment of CIC^10^ and that 5-HT_4_Rs are highly abundant in the GI tract^32^, we explored the fast antidepressant/anxiolytic effects of the 5-HT_4_R agonist prucalopride and its effects on the gut microbiota, but also microbiota-targeting effects of chronic CORT, in the context of the gut-brain axis. Extensive human cohort studies have highlighted significant disruptions in gut microbiota composition associated with neuropsychiatric disorders, such as anxiety disorders and MDD^33^. Specifically, the gut microbiota plays an important role in the gut-brain axis, influencing depression through multiple pathways including the hypothalamic-pituitary-adrenal (HPA) axis. Interestingly, there seems to be a bidirectional link between the gut microbiota and the HPA axis, which can ultimately result in shifts in CORT levels. Stress-induced glucocorticoid release is modulated by the gut microbiota; for example, germ-free (GF) mice display stress-induced exaggerated glucocorticoid levels that can be recovered upon colonization with specific pathogen-free (SPF) feces^34,35^. Corroborating and expanding these findings, a recent paper showed that microbial depletion dysregulates not only glucocorticoid levels but also their circadian rhythmicity^22^. While the role of the gut microbiota on stress response is becoming more and more evident, few studies to date have looked at the effects of CORT administration on the gut microbiota. We took advantage of the chronic CORT stress model to explore the gut-targeting effects of CORT supplementation. Our results revealed specific time-point-dependent changes in microbial composition. At 5 weeks, the Chao1 index for alpha-diversity was decreased by CORT, and the beta-diversity clearly showed two separate clusters for vehicle *versus* CORT mice. Several taxa were also depleted by CORT. At 8 weeks, however, there were no differences in alpha-diversity and the separation between groups on the Bray-Curtis beta-diversity plot was less evident. At this timepoint, some individual changes at the genus level persisted (i.e., a decrease in *Clostridia UCG014* and *Ruminococcus*) and others appeared. A recent study showed that a chronic exposure to CORT via i.p. in male C57BL6/J mice induced not only anxiety- and depressive-like behaviors, but also specific changes in the gut microbiota, such as increases in *Lactobacillus*, *Bacillus*, and *Bifidobacterium*^36^. It is interesting to note that we also reported an increase in *Lactobacillus*, but no similarities were found regarding the other two genera. The CORT administration protocol differed in terms of dose, duration, and route, which may account for variations in microbiota composition observed between studies. Overall, these findings show that CORT supplementation can impact not only circulating levels of CORT, as expected, but also the gut microbiota composition in a duration-dependent manner.

Recent findings emphasize that antidepressants can markedly alter the gut microbiota, with their effects surpassing those associated with the diagnosis of neuropsychiatric disorders alone^37^. Among psychotropic drugs, antidepressants’ ability to target the microbiota has been extensively studied both in vitro and in vivo [for review^38^]. Specifically, the SSRIs escitalopram and fluoxetine showed differential antimicrobial activity in vitro against *E.coli* and *L. rhamnosus*, two bacteria residing in the human gut^39^, but also against *Staphylococcus* and *Enterococcus*^40,41^. Consistent with these findings, it is not surprising to observe a fluoxetine-related effect on the gut microbiota, whether after subchronic or chronic treatment, even though the effect of the CORT model seems to be prevalent. Although SSRIs do not directly target serotonin receptors, they are considered indirect agonists; they might therefore act similarly to prucalopride in shifting 5-HT intestinal transmission and, in turn, microbial composition.

We next focused on prucalopride’s distal effects. While at the subchronic timepoint we mostly observed CORT-induced changes, after chronic administration, despite the absence of changes in global alpha-diversity metrics, we saw a specific CORT-induced depletion in the genus *Ruminococcus* which was restored following prucalopride treatment at both tested doses. Functionally, bacteria belonging to this genus serve to degrade and convert complex polysaccharides into a variety of nutrients for their host^42^. Interestingly, *Ruminococcus* was found to be depleted in rodent models of depression other than the CORT model, including the chronic unpredictable mild stress (CUMS) model^43^, the maternal separation model^44^, and the social defeat (SD) model^45^, suggesting that its depletion might be independent of the causal factor and stable across models. Conversely recent preclinical findings^46,47^ showed that (i) *Ruminococcus* was decreased following conventional antidepressant treatment (fluoxetine, duloxetine) and (ii) introduction in male BALB/c OlaHsd mice of a single *Ruminococcus* species, *R. flavefaciens*, induced cortical transcriptional changes, including the up-regulation of genes related to mitochondrial oxidative phosphorylation and down-regulation of genes associated with neuronal plasticity, which were interpreted as counteracting the antidepressant response. Taken together, these seemingly divergent results raise important questions about the genus *Ruminococcus* and its strain-specific effects on host physiology and antidepressant efficacy. One possibility is that different *Ruminococcu*s strains may exert opposite influences on behavioral outcomes, depending on the pharmacological context and host state (e.g., chronic stress vs. pharmacological antidepressant exposure). Alternatively, prucalopride may engage gut-brain signaling pathways that leverage *Ruminococcus* restoration in a manner distinct from classical monoaminergic antidepressants. In our study, several other taxa were affected by subchronic and chronic treatments, but the observed shifts did not seem to be prucalopride-specific. A limitation of the current design is the absence of fluoxetine- and prucalopride-only groups. This restricts our ability to distinguish drug-specific microbiome effects from those occurring only in the context of chronic CORT exposure. Future studies incorporating these groups are essential to fully tease out treatment-specific versus stress-modulatory effects on the gut microbiota. It is also important to note that this type of grouped housing may introduce microbiome-related cage effects, which is a limitation of the current study. Although our current findings on *Ruminococcus* remain observational and do not supply functional readouts, they provide nonetheless a basis for future research aimed at exploring the potential causal role of this genus in prucalopride-induced fast anxiolytic/antidepressant effects. Importantly, these preclinical observations are supported by clinical evidence. To date, seven clinical studies have reported a depletion of *Ruminococcus* in depressed patients relative to healthy controls, although two studies observed an increase in this genus^48^. Discrepancies may stem from confounding factors such as diet and lifestyle. Notably, one clinical study reported that *Ruminococcus* depletion was specific to “severely depressed” patients^49^, suggesting that its reduction may be linked to disease severity. Taken together, the convergence of preclinical and clinical data highlights *Ruminococcus* as a potentially relevant genus in both depression pathophysiology and antidepressant response, warranting further investigation.

Overall, this study shows concomitant changes in the gut microbiota and behavioral outcomes relevant to depression and anxiety. It is important to highlight certain limitations of this study, some of which could be addressed in future experiments. First, 16S sequencing is limited to the genus levels as compared to a whole genome shotgun sequencing, which would further allow the functional classification of the microbiota and *Ruminococcus*-associated metabolic pathways. Secondly, prucalopride, a drug used to treat CIC, could induce GI side effects like diarrhea in mice, but this was not the case in our experiment. However, deeper measures of intestinal permeability could be useful in future experiments, especially considering that microbiota plays a role in regulating intestinal barrier function^50^ and GI comorbidities can often occur together with psychiatric disorders^51^. The absence of gut motility outcomes represent a limitation that should be addressed in future studies. In addition, prucalopride was administered via osmotic minipumps: the question arises as to whether an oral administration, which directly reaches the GI tract, would differently affect the microbiota of the mice; therefore, comparing different routes of administration would be informative. Finally, isoflurane anesthesia can impact the microbiota composition^52^, a factor that was not accounted for and represents a limitation of the present study. Future studies would be valuable to track within-subject microbial and behavioral dynamics over time, with the complementary goal to evaluate microbial stability.

These findings shed light on an additional mechanism through which prucalopride exerts its effects, highlighting the intricate and dynamic relationship between the gut and the brain. The dual behavioral and microbiota-modulating effects of the 5-HT_4_R agonist prucalopride provide a solid foundation for further research into the therapeutic potential of 5-HT_4_R-targeting molecules in the treatment of MDD. Notably, *Ruminococcus* emerges as a compelling bacterial candidate for exploring the microbiota’s role in mediating prucalopride’s central effects. The twofold benefits of prucalopride in improving both mood disorders and gut health underscore its potential as a novel therapeutic option. Future investigations could pave the way for its broader application in addressing the interconnected challenges of mental health and gastrointestinal well-being.

## Methods

### Animals

Adult male C57BL/6JRj mice were purchased from Janvier Labs (Le Genest-Saint-Isle, France). Mice were 7-8 weeks old and weighed 23–35 g at the beginning of the experiment, were maintained in a room with a controlled temperature (21 ± 1°C) on a 12-h/12-h light/dark cycle and were housed four per cage. Food and water were provided *ad libitum*. Behavioral testing occurred during the light phase. Protocols were approved by the Institutional Animal Care and Use Committee (IACUC) in France (Council directive n° 87-848, October 19, 1987, Ministère de l’Agriculture et de la Forêt, Service Vétérinaire de la Santé et de la Protection Animale, protocol APAFIS n° 39512) as well as with the European directive 2010/63/EU.

### Drugs

Corticosterone [4-pregnen-11b-DIOL-3 20-DIONE 21-hemi-succinate, CORT (35 µg/mL)] purchased from Sigma-Aldrich (Saint-Quentin Fallavier, France) was dissolved in a vehicle [Veh = 0.45% hydroxypropyl-β-cyclodextrin (β-CD); Sigma-Aldrich]. Fluoxetine hydrochloride (Flx = 160 mg/L, equivalent to 18 mg/kg/day) was purchased from Anawa Trading (Zurich, Switzerland) and dissolved in 0.45% β-CD/CORT solution. Prucalopride (4-Amino-5-chloro-2,3-dihydro-N-[1-(3-methoxypropyl)-4-piperidinyl]-7-benzofurancarboxamide, 0.5 mg/kg/day and 1.5 mg/kg/day), dissolved in a mixture of 0.9% saline and 0.5% dimethyl sulfoxide (DMSO), was delivered by osmotic minipumps (28 days minipumps, 2004 model, Alzet, Cupertino, CA) implanted subcutaneously under light isoflurane anesthesia (3% induction and 1% maintenance) (Fig. S1). CORT-treated drinking water was replaced every 3 days to prevent any possible degradation as previously described^7,20^ and maintained throughout the experiment. Control animals (Veh/Veh and CORT/Veh groups) were also implanted with an Alzet minipump (model 2004) delivering 0.9% saline and 0.5% DMSO. CORT and Flx doses were calculated based an average fluid intake of 5 mL/mouse/day^20^. Instead of regular drinking water, mice were exposed for 8 weeks with Veh or CORT in the presence or absence of drugs (fluoxetine or prucalopride) administered during the last four weeks of the CORT protocol. Plasmatic drug levels were measured after subchronic (day 7) and chronic (day 28) treatments (Table S1).

### Behavioral tests

All behavioral tests were conducted during the light phase (7 am–7 pm). The same animal was successively tested in three different behavioral paradigms [Elevated Plus Maze (EPM), the Novelty Suppressed Feeding (NSF) paradigm, and the Splash Test (ST)], from the least to the most aversive after a subchronic (days 3–7) and chronic treatment (days 24–28) with vehicle, fluoxetine or prucalopride (Fig. S1).

#### Elevated plus maze

The EPM is a widely used behavioral assay for rodents and it has been validated to assess the anti-anxiety effects of pharmacological agents^53^. This test was performed as described previously^7^. The maze was a plus-cross-shaped apparatus, with two open arms and two arms, each 45 cm long × 10 cm wide, linked by a central platform, 60 cm above the floor. Mice were individually put in the center of the maze facing an open arm and were allowed to explore the maze for a duration of 5 min. The time spent in the open arms and the number of entries into them were used as indexes of anxiety. The whole set-up was controlled using ANYMAZE version 7.4 video tracking software (Stoelting Co/Vivo-tech, Salon de Provence, France).

#### Novelty-suppressed feeding

The NSF is a conflict test that elicits competing motivations: the drive to eat and the fear of venturing into the center of a brightly lit arena^54^. Testing was performed as previously described^20^. Briefly, the NSF testing apparatus consisted of a plastic box (50 × 50 × 20 cm). The floor of which was covered with approximately 2 cm of wooden bedding and the arena was brightly lit. Mice were food restricted for 24 h prior to testing. At the time of testing, a single pellet of food (regular chow) was placed on a white paper platform in the center of the box. Each animal was placed in a corner of the box, and a stopwatch was immediately started. The NSF test was performed during a 10 min period as previously described^20^. The latency of the mice to begin eating in the arena was recorded. Immediately after the latency was recorded, the food pellet was removed from the arena. The mice were then placed back into their home cages. The latency to eat and the amount of food consumed in 5 min were measured (home cage consumption), followed by an assessment of post-fasting weight.

#### Splash test

This test consisted of squirting about 200 µL of a 10% sucrose solution on the mouse’s back^55^. This procedure induces grooming behaviors, due to the viscosity and palatability of the sucrose. The grooming behavior is sensitive to chronic stress or chronic CORT exposure and antidepressant treatment^7^. The total time spent grooming was recorded for 5 min in the home cage.

#### Emotionality score

The behavioral tests were used to compile behavioral emotionality of the mice (Z-score) as previously described^56^. Briefly, Z-scores are standardized scores (by the group mean and group standard deviation). They indicate how many standard deviations (σ) an observation (x) is above or below the mean of a control group (μ). Z-scores for behavioral measures were first averaged within the test, and then across the test for equal weighting of the three tests comprising the final emotionality score.

### Fecal microbiota composition

#### Feces collection

For feces collection, mice were isolated in plastic boxes. The collection was performed in the morning when mice readily produce feces. Simple handling of the mice in the boxes was sufficient to produce feces. As soon as 3 droppings had been collected, and after a maximum of 10 min of isolation, the mice were returned to their original cages. After collection, feces were weighed and stored at –80 ^°^C.

#### Bacterial DNA extraction

DNA was extracted using the Qiagen QIAmp Fast DNA Stool Mini Kit. The V3-V4 hypervariable region of the 16S rRNA gene was amplified and prepared for sequencing as outlined in the Illumina 16S Metagenomic Sequencing Library protocol. Samples were sequenced at (GenoToul, Toulouse) on the Illumina MiSeq platform using a (2 × 300 base pairs) as previously described by Picher and colleagues^57^. InhibitEX buffer was pre-heated and homogenized to eliminate crystals and safeguard functionality. Elution allowed efficient DNA purification. DNA concentrations were measured by a Nanodrop spectrophotometer.

#### V3-V4 PCR amplification of the 16S gene

The V3-V4 region of the 16S rRNA gene were targeted and amplified using the universal primers: PCR1F_460: 5′ CTITCCCTACACGACGCTCTTCCGATCTACGGRAGGCAGCAG3 ; PCR1R_460: 5′ GGAGITCAGACGTGTGCTCTTCCGATCTTACCAGGGTATCTAATCCT3′ and the conventional PCR protocol for 16S/18S MolTaq enzyme was followed (Alliance Bio Expertise, Breman, Germany). The number of PCR cycles was 30 and the concentration of bacterial DNA used was 50 ng/µL. The amplicons are migrated on % agarose gel and visualized under UV light to verify their quality. The 16S rRNA gene is a conserved phylogenetic marker among prokaryotes and allows the differentiation among the latter in a metagenomic sample, such as feces. The V3-V4 region allows discrimination among bacterial taxa and the calculation of relative abundances^58^.

### Statistical analyses

All statistical tests and *p* values are listed in Supplemental Tables (Tables S2–S7).

#### Behavior analysis

Statistical analyses were performed using Prism Graphpad version 10.2.3. A one-way ANOVA followed by LSD multiple comparison test was employed for the EPM, ST and NSF (except for the survival analysis). The Fisher’s LSD test was chosen for its sensitivity in detecting pairwise differences following a significant ANOVA, particularly in small-sample exploratory analyses. However, follow-up studies with larger sample sizes would be required to confirm these findings. In the NSF, A Kaplan-Meier survival analysis was used due to the lack of normal distribution of data. The Mantel-Cox log-rank test was used to evaluate differences between experimental groups. All probabilities were two-sided with statistical significance set at *p* < 0.05.

#### 16S rRNA sequencing data analysis

Data analysis was conducted using the QIIME2 bioinformatics package (version 2024.10) and the user-friendly software MicrobiomeAnalyst (version 2.0). The average number of reads was 24,418. Samples with less than 10k reads were excluded. Reads were denoised on a sequence base threshold of 240 which corresponded to a quality score ≥ 20, and rarefied to the median frequency of 14k. After quality control, the paired sequences that are 97% similar were clustered into Operational Taxonomic Units (OTUs) de novo. OTUs with low abundances were excluded from downstream analysis (maximum combined abundance <10). Bacterial taxa were assigned by aligning the OTUs to pre-defined sequences in the reference database classifier SILVA (silva-138-99-nb-classifier). Raw count data were transformed using the CLR (centered log ratio) transformation before conducting statistical analysis. Principal component analysis (PCoA), which evaluates the dissimilarity between samples, was performed on CLR values using the Bray-Curtis index and analyzed by a PERMANOVA. Non-parametric tests were used for compositional abundances (e.g., individual taxa, Kruskal-Wallis, and Mann-Whitney tests). To control for the False Discovery Rate (FDR) related to multiple testing, significant features were selected using the Benjamini-Hochberg procedure with a q-value of 0.2. All probabilities were two-sided with statistical significance set at *p* < 0.05.

## Supplementary information

PrucaGM_suppl mat_v10

## Data Availability

The data that support the findings of this study are available from the corresponding author upon reasonable request.

## References

[1] Malhi, G. S. & Mann, J. J. Depression. *Lancet* **392**, 2299–2312 (2018). 30396512 10.1016/S0140-6736(18)31948-2

[2] Gourion, D. Antidepressants and their onset of action: A major clinical, methodological and pronostical issue]. *Encephale* **34**, 73–81 (2008). 18514154 10.1016/j.encep.2007.12.001

[3] Guina, J. & Merrill, B. Benzodiazepines I: Upping the care on downers: The evidence of risks, benefits and alternatives. *J. Clin. Med*. **7**, 17 (2018). 29385731 10.3390/jcm7020017 PMC5852433

[4] Penninx, B. W., Pine, D. S., Holmes, E. A. & Reif, A. Anxiety disorders. *Lancet* **397**, 914–927 (2021). 33581801 10.1016/S0140-6736(21)00359-7 PMC9248771

[5] Samuels, B. A. et al. Serotonin 1A and serotonin 4 receptors: Essential mediators of the neurogenic and behavioral actions of antidepressants. *Neuroscientist* **22**, 26–45 (2016). 25488850 10.1177/1073858414561303 PMC4714598

[6] Faye, C. et al. Rapid anxiolytic effects of RS67333, a serotonin type 4 receptor agonist, and diazepam, a benzodiazepine, are mediated by projections from the prefrontal cortex to the dorsal raphe nucleus. *Biol. Psychiatry* **87**, 514–525 (2020). 31623825 10.1016/j.biopsych.2019.08.009

[7] Mendez-David, I. et al. Rapid anxiolytic effects of a 5-HT4 receptor agonist are mediated by a neurogenesis-independent mechanism. *Neuropsychopharmacology* **39**, 1366–1378 (2014). 24287720 10.1038/npp.2013.332 PMC3988540

[8] Poinsignon, V. et al. The GG genotype of the serotonin 4 receptor genetic polymorphism, rs1345697, is associated with lower remission rates after antidepressant treatment: Findings from the METADAP cohort. *J. Affect Disord.* **299**, 335–343 (2022). 34906639 10.1016/j.jad.2021.12.012

[9] Köhler-Forsberg, K. et al. Serotonin 4 receptor brain binding in major depressive disorder and association with memory dysfunction. *JAMA Psychiatry* **80**, 296–304 (2023). 36753296 10.1001/jamapsychiatry.2022.4539 PMC9909578

[10] Mahajan, R. Prucalopride: A recently approved drug by the food and drug administration for chronic idiopathic constipation. *Int J. Appl Basic Med Res.* **9**, 1–2 (2019). 30820411 10.4103/ijabmr.IJABMR_412_18 PMC6385531

[11] Johnson, D. et al. The 5-hydroxytryptamine4 receptor agonists prucalopride and PRX-03140 increase acetylcholine and histamine levels in the rat prefrontal cortex and the power of stimulated hippocampal oscillations. *J. Pharmacol. Exp. Ther.* **341**, 681–691 (2012). 22408061 10.1124/jpet.112.192351

[12] De Cates, A. N. et al. Association between a selective 5-HT4 receptor agonist and incidence of major depressive disorder: emulated target trial. *Br. J. Psychiatry* **225**, 371–378. 10.1192/bjp.2024.97 PMC7616487 39109752

[13] Chen, B. K. et al. Prophylactic efficacy of 5-HT4R agonists against stress. *Neuropsychopharmacology* **45**, 542–552 (2020). 31600767 10.1038/s41386-019-0540-3 PMC6969048

[14] Makovkina, E., Brody, B. D. & Shaffer, C. Possible association of 5-HT4 receptor agonist prucalopride in a 52-year-old man with an index manic episode. *Bipolar Disord.* **24**, 767–769 (2022). 35950919 10.1111/bdi.13245

[15] Kendig, D. M. & Grider, J. R. Serotonin and colonic motility. *Neurogastroenterol. Motil.* **27**, 899–905 (2015). 26095115 10.1111/nmo.12617 PMC4477275

[16] Terry, N. & Margolis, K. G. Serotonergic mechanisms regulating the GI tract: Experimental evidence and therapeutic relevance. *Handb. Exp. Pharm.* **239**, 319–342 (2017). 10.1007/164_2016_103 PMC5526216 28035530

[17] Cryan, J. F. et al. The microbiota-gut-brain axis. *Physiol. Rev.* **99**, 1877–2013 (2019). 31460832 10.1152/physrev.00018.2018

[18] Cussotto, S., Sandhu, K. V., Dinan, T. G. & Cryan, J. F. The neuroendocrinology of the microbiota-gut-brain axis: A behavioural perspective. *Front Neuroendocrinol.* **51**, 80–101 (2018). 29753796 10.1016/j.yfrne.2018.04.002

[19] Johnson, J. S. et al. Evaluation of 16S rRNA gene sequencing for species and strain-level microbiome analysis. *Nat. Commun.* **10**, 5029 (2019). 31695033 10.1038/s41467-019-13036-1 PMC6834636

[20] David, D. J. et al. Neurogenesis-dependent and -independent effects of fluoxetine in an animal model of anxiety/depression. *Neuron* **62**, 479–493 (2009). 19477151 10.1016/j.neuron.2009.04.017 PMC2759281

[21] Mendez-David, I. et al. A complex relation between levels of adult hippocampal neurogenesis and expression of the immature neuron marker doublecortin. *Hippocampus* **33**, 1075–1093 (2023). 37421207 10.1002/hipo.23568

[22] Tofani, G. S. S. et al. Gut microbiota regulates stress responsivity via the circadian system. *Cell Metab.*10.1016/j.cmet.2024.10.003 (2024) 10.1016/j.cmet.2024.10.003 39504963

[23] Ergang, P. et al. The gut microbiota affects corticosterone production in the murine small intestine. *Int. J. Mol. Sci.* **22**, 4229 (2021). 33921780 10.3390/ijms22084229 PMC8073041

[24] Trivedi, M. H. et al. Evaluation of outcomes with citalopram for depression using measurement-based care in STAR*D: Implications for clinical practice. *Am. J. Psychiatry* **163**, 28–40 (2006). 16390886 10.1176/appi.ajp.163.1.28

[25] Kato, M. & Serretti, A. Review and meta-analysis of antidepressant pharmacogenetic findings in major depressive disorder. *Mol. Psychiatry* **15**, 473–500 (2010). 18982004 10.1038/mp.2008.116

[26] Wong, A., Taylor, D. M., Ashby, K. & Robinson, J. Changing epidemiology of intentional antidepressant drug overdose in Victoria, Australia. *Aust. N. Z. J. Psychiatry* **44**, 759–764 (2010). 20636198 10.3109/00048674.2010.481279

[27] Griffin, C. E., Kaye, A. M., Bueno, F. R. & Kaye, A. D. Benzodiazepine pharmacology and central nervous system–mediated effects. *Ochsner J.* **13**, 214–223 (2013). 23789008 PMC3684331

[28] Taylor, C., Fricker, A. D., Devi, L. A. & Gomes, I. Mechanisms of action of antidepressants: From neurotransmitter systems to signaling pathways. *Cell Signal* **17**, 549–557 (2005). 15683730 10.1016/j.cellsig.2004.12.007 PMC3581018

[29] Lucas, G. et al. Serotonin(4) (5-HT(4)) receptor agonists are putative antidepressants with a rapid onset of action. *Neuron* **55**, 712–725 (2007). 17785179 10.1016/j.neuron.2007.07.041

[30] Pascual-Brazo, J. et al. Modulation of neuroplasticity pathways and antidepressant-like behavioural responses following the short-term (3 and 7 days) administration of the 5-HT4 receptor agonist RS67333. *Int. J. Neuropsychopharmacol.* **15**, 631–643 (2012). 21733238 10.1017/S1461145711000782

[31] Tamburella, A., Micale, V., Navarria, A. & Drago, F. Antidepressant properties of the 5-HT4 receptor partial agonist, SL65.0155: Behavioral and neurochemical studies in rats. *Prog. Neuropsychopharmacol. Biol. Psychiatry* **33**, 1205–1210 (2009). 19596038 10.1016/j.pnpbp.2009.07.001

[32] Yaakob, N. S., Chinkwo, K. A., Chetty, N., Coupar, I. M. & Irving, H. R. Distribution of 5-HT3, 5-HT4, and 5-HT7 receptors along the human colon. *J. Neurogastroenterol. Motil.* **21**, 361 (2015). 26130632 10.5056/jnm14157 PMC4496915

[33] Patel, R. A., Panche, A. N. & Harke, S. N. Gut microbiome-gut brain axis-depression: interconnection. *World J. Biol. Psychiatry* **26**, 1–36 (2025). 39713871 10.1080/15622975.2024.2436854

[34] Lyte, J. M. et al. Gut-brain axis serotonergic responses to acute stress exposure are microbiome-dependent. *Neurogastroenterol. Motil.* **32**, e13881 (2020). 32391630 10.1111/nmo.13881

[35] Sudo, N. et al. Postnatal microbial colonization programs the hypothalamic–pituitary–adrenal system for stress response in mice. *J. Physiol.* **558**, 263–275 (2004). 15133062 10.1113/jphysiol.2004.063388 PMC1664925

[36] Wang, G. et al. Gut microbiota dysbiosis-mediated ceramides elevation contributes to corticosterone-induced depression by impairing mitochondrial function. *npj Biofilms Microbiomes* **10**, 111 (2024). 39468065 10.1038/s41522-024-00582-w PMC11519513

[37] Dilmore, A. H. et al. Medication use is associated with distinct microbial features in anxiety and depression. *Mol. Psychiatry* 1–13 10.1038/s41380-024-02857-2 (2025). 10.1038/s41380-024-02857-2 PMC12092254 39794490

[38] Cussotto, S., Clarke, G., Dinan, T. G. & Cryan, J. F. Psychotropics and the microbiome: A chamber of secrets. *Psychopharmacol. (Berl.)* **236**, 1411–1432 (2019). 10.1007/s00213-019-5185-8 PMC6598948 30806744

[39] Cussotto, S. et al. Differential effects of psychotropic drugs on microbiome composition and gastrointestinal function. *Psychopharmacol. (Berl.)* **236**, 1671–1685 (2019). 10.1007/s00213-018-5006-5 30155748

[40] Ayaz, M. et al. Sertraline enhances the activity of antimicrobial agents against pathogens of clinical relevance. *J. Biol. Res. (Thessalon.)* **22**, 4 (2015). 26029671 10.1186/s40709-015-0028-1 PMC4449573

[41] Coban, A. Y., Tanriverdi Cayci, Y., Keleş Uludağ, S. & Durupinar, B. Investigation of antibacterial activity of sertralin]. *Mikrobiyol. Bul.* **43**, 651–656 (2009). 20084919

[42] La Reau, A. J. & Suen, G. The Ruminococci: Key symbionts of the gut ecosystem. *J. Microbiol* **56**, 199–208 (2018). 29492877 10.1007/s12275-018-8024-4

[43] Li, Y. & Liu, P. Characteristics of oral-gut microbiota in model rats with CUMS-induced depression. *Neuropsychiatr. Dis. Treat.* **20**, 221–232 (2024). 38344423 10.2147/NDT.S448940 PMC10854232

[44] Kemp, K. M., Colson, J., Lorenz, R. G., Maynard, C. L. & Pollock, J. S. Early life stress in mice alters gut microbiota independent of maternal microbiota inheritance. *Am. J. Physiol. Regul. Integr. Comp. Physiol.* **320**, R663–R674 (2021). 33655759 10.1152/ajpregu.00072.2020 PMC8163610

[45] McGaughey, K. D. et al. Relative abundance of Akkermansia spp. and other bacterial phylotypes correlates with anxiety- and depressive-like behavior following social defeat in mice. *Sci. Rep.* **9**, 3281 (2019). 30824791 10.1038/s41598-019-40140-5 PMC6397238

[46] Liu, L. et al. Gut microbiota and its metabolites in depression: From pathogenesis to treatment. *eBioMedicine* **90**, 104527 (2023). 36963238 10.1016/j.ebiom.2023.104527 PMC10051028

[47] Lukić, I. et al. Antidepressants affect gut microbiota and Ruminococcus flavefaciens is able to abolish their effects on depressive-like behavior. *Transl. Psychiatry* **9**, 133 (2019). 30967529 10.1038/s41398-019-0466-x PMC6456569

[48] Gao, M. et al. Gut microbiota composition in depressive disorder: a systematic review, meta-analysis, and meta-regression. *Transl. Psychiatry* **13**, 379 (2023). 38065935 10.1038/s41398-023-02670-5 PMC10709466

[49] Hu, X. et al. Changes of gut microbiota reflect the severity of major depressive disorder: a cross sectional study. *Transl. Psychiatry* **13**, 1–9 (2023). 37117202 10.1038/s41398-023-02436-z PMC10147706

[50] Ghosh, S., Whitley, C. S., Haribabu, B. & Jala, V. R. Regulation of intestinal barrier function by microbial metabolites. *Cell Mol. Gastroenterol. Hepatol.* **11**, 1463–1482 (2021). 33610769 10.1016/j.jcmgh.2021.02.007 PMC8025057

[51] Person, H. & Keefer, L. Psychological comorbidity in gastrointestinal diseases: Update on the brain-gut-microbiome axis. *Prog. Neuropsychopharmacol. Biol. Psychiatry* **107**, 110209 (2021). 33326819 10.1016/j.pnpbp.2020.110209 PMC8382262

[52] Serbanescu, M. A. et al. General anesthesia alters the diversity and composition of the intestinal microbiota in mice. *Anesth. Analg.* **129**, e126–e129 (2019). 30489316 10.1213/ANE.0000000000003938 PMC9717490

[53] Walf, A. A. & Frye, C. A. The use of the elevated plus maze as an assay of anxiety-related behavior in rodents. *Nat. Protoc.* **2**, 322–328 (2007). 17406592 10.1038/nprot.2007.44 PMC3623971

[54] Francois, M., Canal Delgado, I., Shargorodsky, N., Leu, C.-S. & Zeltser, L. Assessing the effects of stress on feeding behaviors in laboratory mice. *eLife* **11**, e70271 (2022). 35167441 10.7554/eLife.70271 PMC8846584

[55] Bouguiyoud, N. et al. Anxiety and depression assessments in a mouse model of congenital blindness. *Front. Neurosci*. **15**, 807434 (2022). 10.3389/fnins.2021.807434 PMC8816321 35126047

[56] Guilloux, J.-P., Seney, M., Edgar, N. & Sibille, E. Integrated behavioral z-scoring increases the sensitivity and reliability of behavioral phenotyping in mice: Relevance to emotionality and sex. *J. Neurosci. Methods* **197**, 21–31 (2011). 21277897 10.1016/j.jneumeth.2011.01.019 PMC3086134

[57] Pichler, M. et al. A 16S rRNA gene sequencing and analysis protocol for the Illumina MiniSeq platform. *Microbiologyopen* **7**, e00611 (2018). 29575567 10.1002/mbo3.611 PMC6291791

[58] Shahi, S. K., Zarei, K., Guseva, N. V. & Mangalam, A. K. Microbiota analysis using two-step PCR and next-generation 16S rRNA gene sequencing. *J. Vis. Exp.*10.3791/59980 (2019). 10.3791/59980 PMC6945761 31680682

