# Supplementary material for: Prucalopride, a serotonin type 4 receptor agonist, induces fast anxiolytic/antidepressant effects and concomitant changes in the gut microbiota
Source: NPJ Biofilms Microbiomes. 2026 Feb 4;12:62. doi: 10.1038/s41522-026-00928-6 (PMC12979823; doi:10.1038/s41522-026-00928-6)
Supplement: Supplementary file 1 — PrucaGM_suppl mat_v10 [file 41522_2026_928_MOESM1_ESM.pdf]

SUPPLEMENTAL DATA

Prucalopride, a Serotonin Type 4 Receptor Agonist, Induces Fast Anxiolytic/Antidepressant Effects and Concomitant Changes in the Gut Microbiota

Sofia Cussotto[1], Salma R. Abdennebi[1], Isabelle Etting[2,3], Christine A. Denny[4], René Hen[4], Romain Colle[5,6], Emmanuelle Corruble[5,6], Jean-Claude Alvarez[2,3], Denis J. David[1,#], Indira Mendez-David[1]

[1] Université Paris-Saclay, UVSQ, Centre de recherche en Epidémiologie et Santé des Populations (CESP), UMR 1018, CESP-Inserm, Team Moods, Faculté de Pharmacie, Bâtiment Henri Moissan, Orsay, France
[2] Department of Pharmacology and Toxicology, Raymond Poincaré Hospital, GHU AP-HP, UVSQ/Paris-Saclay, Garches, France
[3] Inserm U-1018, CESP, Team MOODS, Paris-Saclay/Versailles University, Garches, France
[4] Department of Psychiatry, Columbia University Irving Medical Center, New York, New York; Division of Systems Neuroscience, Area Neuroscience, Research Foundation for Mental Hygiene, Inc./New York State Psychiatric Institute, New York, New York
[5] Université Paris-Saclay, Centre de recherche en Epidémiologie et Santé des Populations (CESP), MOODS UMR1018, CESP-Inserm, Team Moods, Faculté de Médecine, Le Kremin-Bicêtre, France
[6] Service Hospitalo-Universitaire de Psychiatrie, Hôpital de Bicêtre, Hôpitaux Universitaires Paris-Saclay, Assistance Publique-Hôpitaux de Paris, Le Kremlin-Bicêtre, France

---

I. SUPPLEMENTAL EXPERIMENTAL PROCEDURES

Corticosterone, prucalopride, fluoxetine and its main metabolite levels in mouse plasma

Blood was collected from unanesthetized mice as previously described.[1] In compliance with the laboratory animal care guidelines, 5 mm point size sterile lancets (MediPoint, Mineola, NY, USA) were used to puncture the location where the orbital vein and the submandibular vein join to form the jugular vein. A light pressure with dry gauze was applied to the punctured area for hemostasis. Approximately 0.4 mL of blood per mouse was collected in K_3EDTA tubes with a submandibular bleeding procedure and centrifuged at 3000 rpm for 10 min (at 4 °C) for separation of plasma. Samples were stored at -80 °C until assayed.

Prucalopride (0.5 and 1.5 mg/kg/day; $Pruca_{0.5}$; $Pruca_{1.5}$), fluoxetine (Flx), its main metabolite norfluoxetine and corticosterone (CORT) were quantified in mouse plasma using liquid chromatography coupled to tandem mass spectrometry detection (LC-MS/MS) using corticosterone-d8 as internal standard (IS). Briefly, to 50 µL of plasma,10 µL of IS solution at 5 mg/L and 200 µL of methanol were added. After agitation, the mixture was incubated 10 min at 4°C, then centrifuged 10 min and supernatant was evaporated until dryness. Residue was reconstituted in 100 µL of a mixture 2 mmol/L formate ammonium +0.1% formic acid and acetonitrile (50/50 v/v). 10 µL were directly injected in a Vanquish chromatographic system coupled to a TSQ Altis triple quadrupole mass spectrometer (ThermoFischer Scientific, Les Ulis, France). Chromatographic separation was performed on a Hypersil Gold PFP column (100×2.1 mm, 1.9 µm) protected by a suitable pre-column. The mobile phase consisted of a gradient of 2 mmol/L formate ammonium, 0.1% formic acid (A) and acetonitrile (B). The gradient started at 30% B and increased to 90% over 10 minutes.

It stabilized at 90% B for 1 minute, then returned to 30%. The flow rate was 0.4 mL/min. Detection was performed in positive electrospray mode.

Data was collected in selected reaction monitoring mode using the transition:

- m/z 368.3 → m/z 196.1 and m/z 279.1 for prucalopride
- m/z 347.3 → m/z 293.2 and m/z 311.2 for corticosterone
- m/z 310.2 → m/z 44.2 and m/z 42.2 for fluoxetine
- m/z 296.1 → m/z 259.1 and m/z 134.0 for norfluoxetine
- m/z 355.3 → m/z 319.2 and m/z 337.3 for corticosterone-d8 (internal standard)

The method was linear between 1 ng/mL and 1000 ng/mL for all compounds. Intra and inter-day precisions of the method were all <15% and accuracies between 90% and 110%. Limits of quantification were 1 ng/mL for all compounds.

Reference

1. Mendez-David, I. *et al.* A method for biomarker measurements in peripheral blood mononuclear cells isolated from anxious and depressed mice: β-arrestin 1 protein levels in depression and treatment. *Front. Pharmacol.* **4**, (2013).

II. SUPPLEMENTAL TABLES

Table S1. Drugs plasma levels after 7 and 28 days of treatment in the corticosterone model of anxiety and depression.

Group	Molecule	Subchronic (7 days)		Chronic (28 days)	
		Mean (ng/mL)	SEM (n)	Mean (ng/mL)	SEM (n)
CORT/Flx	Fluoxetine	777.0	185.6 (6)	1325.0	163.1 (6)
	Norfluoxetine	1425.0	190.4 (6)	1727.0	52.8 (6)
CORT/Pruca$_{0.5}$	Prucalopride	2.3	0.3 (6)	2.2	0.4 (6)
CORT/Pruca$_{1.5}$	Prucalopride	4.0	0.4 (6)	5.7	0.3 (6)

Table S2. Complete statistical summary for behavioral and emotional data following a subchronic treatment of 7 days (related to Figure 1A-G).

Behavior	Outcome	Statistical Test	Comparison	Statistics	df	*p*
Elevated Plus Maze	Time in the OAs (Fig. 1A)	One-way ANOVA	Factor treatment	F=8.98	4, 37	<0.0001 ***
		LSD Post-hoc	Veh/Veh *vs* CORT/Veh			0.0032 **
			Veh/Veh *vs* CORT/Flx			0.0036 **
			Veh/Veh *vs* CORT/$Pruca_{0.5}$			0.3334
			Veh/Veh *vs* CORT/$Pruca_{1.5}$			0.1634
			CORT/Veh *vs* CORT/Flx			0.9088
			CORT/Veh *vs* CORT/$Pruca_{0.5}$			<0.0001 ***
			CORT/Veh *vs* CORT/$Pruca_{1.5}$			0.0477 *
			CORT/Flx *vs* CORT/$Pruca_{0.5}$			<0.0001 ***
			CORT/Flx *vs* CORT/$Pruca_{1.5}$			0.0545
			CORT/$Pruca_{0.5}$ *vs* CORT/$Pruca_{1.5}$			0.0072 **
	Entries in the Oas (Fig. 1B)	One-way ANOVA	Factor treatment	F=5.69	4, 37	0.0011 *
		LSD Post-hoc	Veh/Veh *vs* CORT/Veh			0.0002 ***
			Veh/Veh *vs* CORT/Flx			0.0006 ***
			Veh/Veh *vs* CORT/$Pruca_{0.5}$			0.0928
			Veh/Veh *vs* CORT/$Pruca_{1.5}$			0.0331 *
			CORT/Veh *vs* CORT/Flx			0.6105
			CORT/Veh *vs* CORT/$Pruca_{0.5}$			0.0082 **
			CORT/Veh *vs* CORT/$Pruca_{1.5}$			0.0330 *
			CORT/Flx *vs* CORT/$Pruca_{0.5}$			0.0242 *
			CORT/Flx *vs* CORT/$Pruca_{1.5}$			0.0870
			CORT/$Pruca_{0.5}$ *vs* CORT/$Pruca_{1.5}$			0.5670
	Ratio Amb. Dist. OA/Total (Fig. 1C)	One-way ANOVA	Factor treatment	F=7.17	4, 37	0.0002 **
		LSD Post-hoc	Veh/Veh *vs* CORT/Veh			0.0025 **
			Veh/Veh *vs* CORT/Flx			0.0030 **
			Veh/Veh *vs* CORT/$Pruca_{0.5}$			0.8249
			Veh/Veh *vs* CORT/$Pruca_{1.5}$			0.2741
			CORT/Veh *vs* CORT/Flx			0.8876
			CORT/Veh *vs* CORT/$Pruca_{0.5}$			0.0002 ***
			CORT/Veh *vs* CORT/$Pruca_{1.5}$			0.0162 *
			CORT/Flx *vs* CORT/$Pruca_{0.5}$			0.0002 ***
			CORT/Flx *vs* CORT/$Pruca_{1.5}$			0.0194 *
			CORT/$Pruca_{0.5}$ *vs* CORT/$Pruca_{1.5}$			0.1233
Splash Test	Grooming duration (Fig. 1D)	One-way ANOVA	Factor treatment	F=7.63	4, 37	0.0001 ***
		LSD Post-hoc	Veh/Veh *vs* CORT/Veh			0.0013 **
			Veh/Veh *vs* CORT/Flx			0.2218
			Veh/Veh *vs* CORT/$Pruca_{0.5}$			0.3032
			Veh/Veh *vs* CORT/$Pruca_{1.5}$			0.2696
			CORT/Veh *vs* CORT/Flx			0.0096 **
			CORT/Veh *vs* CORT/$Pruca_{0.5}$			<0.0001 ***
			CORT/Veh *vs* CORT/$Pruca_{1.5}$			0.0085 **
			CORT/Flx *vs* CORT/$Pruca_{0.5}$			0.0092 **
			CORT/Flx *vs* CORT/$Pruca_{1.5}$			0.9046
			CORT/$Pruca_{0.5}$ *vs* CORT/$Pruca_{1.5}$			0.0146 *
Novelty Suppressed Feeding	Latency to Feed (Fig. 1E-F)	Kaplan-Meier Survival	Factor treatment		4	0.0002 ***
		One-way ANOVA	Factor treatment	F=6.31	4, 37	0.0006 ***
		LSD Post-hoc	Veh/Veh *vs* CORT/Veh			0.0003 ***
			Veh/Veh *vs* CORT/Flx			0.0571
			Veh/Veh *vs* CORT/$Pruca_{0.5}$			0.6063
			Veh/Veh *vs* CORT/$Pruca_{1.5}$			0.4115
			CORT/Veh *vs* CORT/Flx			0.0169 *
			CORT/Veh *vs* CORT/$Pruca_{0.5}$			0.0002 ***
			CORT/Veh *vs* CORT/$Pruca_{1.5}$			0.0006 ***
			CORT/Flx *vs* CORT/$Pruca_{0.5}$			0.0956
			CORT/Flx *vs* CORT/$Pruca_{1.5}$			0.1908
			CORT/$Pruca_{0.5}$ *vs* CORT/$Pruca_{1.5}$			0.7151
All	Emotionality score (Fig. 1G)	One-way ANOVA	Factor treatment	F=12.45	4, 37	<0.0001 ***
		LSD Post-hoc	Veh/Veh *vs* CORT/Veh			<0.0001 ***
			Veh/Veh *vs* CORT/Flx			0.0296 *
			Veh/Veh *vs* CORT/$Pruca_{0.5}$			0.6842
			Veh/Veh *vs* CORT/$Pruca_{1.5}$			0.0251 *
			CORT/Veh *vs* CORT/Flx			0.0013**
			CORT/Veh *vs* CORT/$Pruca_{0.5}$			<0.0001 ***
			CORT/Veh *vs* CORT/$Pruca_{1.5}$			0.0023 **
			CORT/Flx *vs* CORT/$Pruca_{0.5}$			0.0028 **
			CORT/Flx *vs* CORT/$Pruca_{1.5}$			0.8927
			CORT/$Pruca_{0.5}$ *vs* CORT/$Pruca_{1.5}$			0.0025 **

Abbreviations: *Amb. Dist.* ambulatory distance; *CORT* corticosterone; *df* degrees of freedom; *Flx* fluoxetine; *OA* open arm; *Pruca* prucalopride; *Veh* vehicle.

Table S3. Complete statistical summary for behavioral and emotional data following a subchronic treatment of 28 days (related to Figure 1H-N).

Behavior	Outcome	Statistical Test	Comparison	Statistics	df	*p*
**Elevated Plus Maze**	**Time in the OAs (Fig. 1H)**	One-way ANOVA	Factor treatment	F=8.20	4, 36	<0.0001 ***
		LSD Post-hoc	Veh/Veh *vs* CORT/Veh			<0.0001 ***
			Veh/Veh *vs* CORT/Flx			0.0012 **
			Veh/Veh *vs* CORT/$Pruca_{0.5}$			<0.0001 ***
			Veh/Veh *vs* CORT/$Pruca_{1.5}$			0.0008 ***
			CORT/Veh *vs* CORT/Flx			0.0195 *
			CORT/Veh *vs* CORT/$Pruca_{0.5}$			0.1766
			CORT/Veh *vs* CORT/$Pruca_{1.5}$			0.0270 *
			CORT/Flx *vs* CORT/$Pruca_{0.5}$			0.2929
			CORT/Flx *vs* CORT/$Pruca_{1.5}$			0.8895
			CORT/$Pruca_{0.5}$ *vs* CORT/$Pruca_{1.5}$			0.3599
	**Entries in the Oas (Fig. 1I)**	One-way ANOVA	Factor treatment	F=5.68	4, 36	0.0012 *
		LSD Post-hoc	Veh/Veh *vs* CORT/Veh			<0.0001 ***
			Veh/Veh *vs* CORT/Flx			0.0172 *
			Veh/Veh *vs* CORT/$Pruca_{0.5}$			0.0007 ***
			Veh/Veh *vs* CORT/$Pruca_{1.5}$			0.0082 **
			CORT/Veh *vs* CORT/Flx			0.0216 *
			CORT/Veh *vs* CORT/$Pruca_{0.5}$			0.3342
			CORT/Veh *vs* CORT/$Pruca_{1.5}$			0.0480 *
			CORT/Flx *vs* CORT/$Pruca_{0.5}$			0.1631
			CORT/Flx *vs* CORT/$Pruca_{1.5}$			0.7240
			CORT/$Pruca_{0.5}$ *vs* CORT/$Pruca_{1.5}$			0.2927
	**Ratio Amb. Dist. OA/Total (Fig. 1J)**	One-way ANOVA	Factor treatment	F=5.66	4, 36	0.0012 *
		LSD Post-hoc	Veh/Veh *vs* CORT/Veh			0.0001 ***
			Veh/Veh *vs* CORT/Flx			0.0421 *
			Veh/Veh *vs* CORT/$Pruca_{0.5}$			0.0022 **
			Veh/Veh *vs* CORT/$Pruca_{1.5}$			0.0764
			CORT/Veh *vs* CORT/Flx			0.0121 *
			CORT/Veh *vs* CORT/$Pruca_{0.5}$			0.2228
			CORT/Veh *vs* CORT/$Pruca_{1.5}$			0.0052 **
			CORT/Flx *vs* CORT/$Pruca_{0.5}$			0.1699
			CORT/Flx *vs* CORT/$Pruca_{1.5}$			0.7402
			CORT/$Pruca_{0.5}$ *vs* CORT/$Pruca_{1.5}$			0.0913
**Splash Test**	**Grooming duration (Fig. 1K)**	One-way ANOVA	Factor treatment	F=15,42	4, 36	<0.0001 ***
		LSD Post-hoc	Veh/Veh *vs* CORT/Veh			0.0004 ***
			Veh/Veh *vs* CORT/Flx			0.0845
			Veh/Veh *vs* CORT/$Pruca_{0.5}$			0.1229
			Veh/Veh *vs* CORT/$Pruca_{1.5}$			0.1445
			CORT/Veh *vs* CORT/Flx			<0.0001 ***
			CORT/Veh *vs* CORT/$Pruca_{0.5}$			<0.0001 ***
			CORT/Veh *vs* CORT/$Pruca_{1.5}$			0.0076 **
			CORT/Flx *vs* CORT/$Pruca_{0.5}$			0.8195
			CORT/Flx *vs* CORT/$Pruca_{1.5}$			0.0004 ***
			CORT/$Pruca_{0.5}$ *vs* CORT/$Pruca_{1.5}$			0.0009 ***
**Novelty Suppressed Feeding**	**Latency to Feed (Fig. 1L-M)**	Kaplan-Meier Survival	Factor treatment		4	<0.0001 ***
		One-way ANOVA	Factor treatment	F=19.38	4, 36	<0.0001 ***
		LSD Post-hoc	Veh/Veh *vs* CORT/Veh			<0.0001 ***
			Veh/Veh *vs* CORT/Flx			0.5919
			Veh/Veh *vs* CORT/$Pruca_{0.5}$			0.0364 *
			Veh/Veh *vs* CORT/$Pruca_{1.5}$			0.0400 *
			CORT/Veh *vs* CORT/Flx			<0.0001 ***
			CORT/Veh *vs* CORT/$Pruca_{0.5}$			<0.0001 ***
			CORT/Veh *vs* CORT/$Pruca_{1.5}$			<0.0001 ***
			CORT/Flx *vs* CORT/$Pruca_{0.5}$			0.0614
			CORT/Flx *vs* CORT/$Pruca_{1.5}$			0.0680
			CORT/$Pruca_{0.5}$ *vs* CORT/$Pruca_{1.5}$			0.9607
**All**	**Emotionality score (Fig. 1N)**	One-way ANOVA	Factor treatment	F=26.63	4, 36	<0.0001 ***
		LSD Post-hoc	Veh/Veh *vs* CORT/Veh			<0.0001 ***
			Veh/Veh *vs* CORT/Flx			0.4832
			Veh/Veh *vs* CORT/$Pruca_{0.5}$			0.0265 *
			Veh/Veh *vs* CORT/$Pruca_{1.5}$			0.0057 **
			CORT/Veh *vs* CORT/Flx			<0.0001 ***
			CORT/Veh *vs* CORT/$Pruca_{0.5}$			<0.0001 ***
			CORT/Veh *vs* CORT/$Pruca_{1.5}$			<0.0001 ***
			CORT/Flx *vs* CORT/$Pruca_{0.5}$			0.0654
			CORT/Flx *vs* CORT/$Pruca_{1.5}$			0.0121 *
			CORT/$Pruca_{0.5}$ *vs* CORT/$Pruca_{1.5}$			0.4623

Abbreviations: *Amb. Dist.* ambulatory distance; *CORT* corticosterone; *df* degrees of freedom; *Flx* fluoxetine; *OA* open arm; *Pruca* prucalopride; *Veh* vehicle.

Table S4A. Kruskal-Wallis statistical summary for microbiota data following a subchronic treatment of 7 days.

Level	Name	Kruskal-Wallis *p*
**Phylum**	Proteobacteria	0.1988
	Desulfobacterota	0.2761
	Actinobacteriota	0.2827
	Firmicutes	0.5599
	Bacteroidota	0.6274
**Family**	Lactobacillaceae	0.1469
	Monoglobaceae	0.1613
	Sutterellaceae	0.1669
	Butyricicoccaceae	0.1977
	Marinifilaceae	0.2213
	Ruminococcaceae	0.2318
	Oscillospiraceae	0.2359
	Eubacterium_coprostanoligenes_group	0.2434
	Clostridia_vadinBB60_group	0.2743
	Desulfovibrionaceae	0.2761
	Eggerthellaceae	0.2827
	Prevotellaceae	0.3223
	Muribaculaceae	0.3308
	Rikenellaceae	0.3684
	Ruminococcaceae UCG_010	0.39
	Lachnospiraceae	0.6098
	Hungateiclostridiaceae	0.6475
	Anaerovoracaceae	0.8417
	Bacteroidaceae	0.8522
	Christensenellaceae	0.8675
	Tannerellaceae	0.9021
	Peptococcaceae	0.9242
**Genus**	*UBA1819*	9.728E-06
	Clostridia_UCG_014	0.0008897
	Ruminococcus	0.005734
	Intestinimonas	0.006296
	Incertae_Sedis	0.007222
	Alloprevotella	0.02736
	Muribaculum	0.04061
	Clostridia _UCG_009	0.1073
	Clostridium ASF356	0.1215
	Lachnospiraceae_FCS020_group	0.133
	Lactobacillus HT002	0.1392
	Lachnispiraceae bacterium A2	0.1408
	Monoglobus	0.1613
	Parasutterella	0.1669
	Lactobacillus	0.1894
	Odoribacter	0.2213
	Lachnospiraceae_UCG_001	0.2278
	Alistipes	0.2306
	Eubacterium_coprostanoligenes_group	0.2434
	Clostridia_vadinBB60_group	0.2743
	Harryflintia	0.2809
	Enterorhabdus	0.2827
	Tuzzerella	0.2984
	Colidextribacter	0.3369
	Oscillibacter	0.3646
	Not_Assigned	0.3686
	Muribaculaceae_genus	0.3714
	uncultured	0.3819
	NK4A214_group	0.387
	Ruminococcaceae UCG_010	0.39
	Anaerotruncus	0.4176
	Bilophila	0.4245
	Butyricicoccus	0.438
	Butyricicoccus	0.438
	Ruminococcaceae UCG_005	0.5487
	Ligilactobacillus	0.5963
	Eubacterium_xylanophilum_group	0.6204
	Eubacterium_xylanophilum_group	0.6204
	Rikenellaceae_RC9_gut_group	0.6452
	Clostridium_GCA_900066575	0.6727
	Marvinbryantia	0.7046
	Lachnospiraceae_NK4A136_group	0.7969
	Eubacterium_nodatum_group	0.8417
	Bacteroides	0.8522
	Prevotellaceae_UCG_001	0.8611
	Peptococcus	0.8643

Taxa highlighted in blue can further undergo a Mann-Whitney test (see Table S4B).

Table S4B. Mann-Whitney statistical summary for microbiota data following a subchronic treatment of 7 days.

Level	Comparison	Name	Mann-Whitney *p*	Rank	Adjusted *q*
Genus	Veh/Veh *vs* CORT/Veh	*UBA1819*	0.001399	1	0.028571429
		Clostridia_UCG_014	0.001399	2	0.057142857
		Ruminococcus	0.001399	3	0.085714286
		Intestinimonas	0.002797	4	0.114285714
		Incertae_Sedis	0.002797	5	0.142857143
		Muribaculum	0.075524	6	0.171428571
		Alloprevotella	0.604196	7	0.2
	Veh/Veh *vs* CORT/Flx	*UBA1819*	0.000999	1	0.028571429
		Clostridia_UCG_014	0.001998	2	0.057142857
		Intestinimonas	0.001998	3	0.085714286
		Incertae_Sedis	0.001998	4	0.114285714
		Muribaculum	0.001998	5	0.142857143
		Alloprevotella	0.105894	6	0.171428571
		Ruminococcus	0.132867	7	0.2
	Veh/Veh *vs* CORT/Pruca$_{0.5}$	*UBA1819*	0.001998	1	0.02857143
		Clostridia_UCG_014	0.003996	2	0.05714286
		Incertae_Sedis	0.003996	3	0.08571429
		Muribaculum	0.007992	4	0.11428571
		Intestinimonas	0.011988	5	0.14285714
		Ruminococcus	0.140859	6	0.17142857
		Alloprevotella	0.945055	7	0.2
	Veh/Veh *vs* CORT/Pruca$_{1.5}$	*UBA1819*	0.001399	1	0.028571429
		Clostridia_UCG_014	0.006993	2	0.057142857
		Incertae_Sedis	0.012587	3	0.085714286
		Intestinimonas	0.016783	4	0.114285714
		Ruminococcus	0.023776	5	0.142857143
		Muribaculum	0.148252	6	0.171428571
		Alloprevotella	0.604196	7	0.2
	CORT/Veh *vs* CORT/Flx	*Clostridia_UCG_014*	0.007859	1	0.028571429
		Ruminococcus	0.010836	2	0.057142857
		Alloprevotella	0.1333	3	NA
		Intestinimonas	0.442724	4	NA
		Incertae_Sedis	0.512384	5	NA
		Muribaculum	0.548962	6	NA
		UBA1819	>0.999999	7	NA
	CORT/Veh *vs* CORT/Pruca$_{0.5}$	*Clostridia_UCG_014*	0.001147	1	0.028571429
		Ruminococcus	0.003096	2	0.057142857
		Alloprevotella	0.188378	3	0.085714286
		Intestinimonas	0.346273	4	0.114285714
		Incertae_Sedis	0.597523	5	0.142857143
		Muribaculum	0.842105	6	0.171428571
		UBA1819	>0.999999	7	0.2
	CORT/Veh *vs* CORT/Pruca$_{1.5}$	*Clostridia_UCG_014*	0.029412	1	0.028571429
		Ruminococcus	0.082353	2	NA
		Intestinimonas	0.359276	3	NA
		Muribaculum	0.436281	4	NA
		Alloprevotella	0.450596	5	NA
		Incertae_Sedis	0.470588	6	NA
		UBA1819	>0.999999	7	NA
	CORT/Flx *vs* CORT/Pruca$_{0.5}$	*Alloprevotella*	0.001505	1	0.02857143
		Intestinimonas	0.075851	2	0.05714286
		Clostridia_UCG_014	0.388772	3	0.08571429
		Muribaculum	0.630529	4	0.11428571
		Incertae_Sedis	0.930341	5	0.14285714
		UBA1819	>0.999999	6	0.17142857
		Ruminococcus	>0.999999	7	0.2
	CORT/Flx *vs* CORT/Pruca$_{1.5}$	*Alloprevotella*	0.010132	1	0.02857143
		Muribaculum	0.065254	2	0.05714286
		Intestinimonas	0.075851	3	0.08571429
		Ruminococcus	0.470783	4	0.11428571
		Clostridia_UCG_014	0.967492	5	0.14285714
		UBA1819	>0.999999	6	0.17142857
		Incertae_Sedis	>0.999999	7	0.2
	CORT/Pruca$_{0.5}$ *vs* CORT/Pruca$_{1.5}$	*Muribaculum*	0.211024	1	NA
		Ruminococcus	0.351209	2	NA
		Clostridia_UCG_014	0.528481	3	NA
		Incertae_Sedis	0.651703	4	NA
		Alloprevotella	0.678029	5	NA
		Intestinimonas	0.99318	6	NA
		UBA1819	>0.999999	7	NA

The largest *p* value that has $p<q$ is significant and all of the *p* values smaller than it are also significant. Highlighted in red are the taxa that maintain significance after the Benjamini-Hochberg correction. $q=(i/m)Q$ where *i* is the rank; *m* is the total number of tests; and *Q* is the false discovery rate (0.2).

Table S5A. Kruskal-Wallis statistical summary analysis for microbiota data following a chronic treatment of 28 days.

Level	Name	Kruskal-Wallis *p*
**Phylum**	Firmicutes	0.125
	Proteobacteria	0.1353
	Bacteroidota	0.1665
	Desulfobacterota	0.1736
	Actinobacteriota	0.958
**Family**	Tannerellaceae	0.0101
	Rikenellaceae	0.01775
	Lactobacillaceae	0.02092
	Prevotellaceae	0.03326
	Eubacterium_coprostanoligenes_group	0.04955
	Clostridia_vadinBB60_group	0.08159
	Marinifilaceae	0.08874
	Lachnospiraceae	0.09403
	Clostridia_UCG_014	0.1315
	uncultured	0.1428
	Monoglobaceae	0.1547
	Desulfovibrionaceae	0.1736
	Bacteroidaceae	0.1788
	Oscillospiraceae	0.2101
	Sutterellaceae	0.2538
	Muribaculaceae	0.2928
	Ruminococcaceae	0.3481
	Anaerovoracaceae	0.4189
	Ruminococcaceae UCG_010	0.5329
	Hungateiclostridiaceae	0.6174
	Christensenellaceae	0.6768
	Eggerthellaceae	0.6768
	Peptococcaceae	0.7963
	Butyricicoccaceae	0.8179
**Genus**	*UBA1819*	0.001388
	Ruminococcus	0.004799
	Lactobacillus	0.01468
	Alistipes	0.04736
	Eubacterium_coprostanoligenes_group	0.04955
	Prevotellaceae_UCG_001	0.05127
	Clostridia_vadinBB60_group	0.08159
	Odoribacter	0.08874
	uncultured	0.1056
	Clostridia_UCG_014	0.1315
	Alloprevotella	0.1339
	Lachnospiraceae_NK4A136_group	0.134
	Anaerotruncus	0.1452
	Not_Assigned	0.1458
	Clostridium ASF356	0.1523
	Monoglobus	0.1547
	Ligilactobacillus	0.1688
	Eubacterium_xylanophilum_group	0.1728
	Bacteroides	0.1788
	Lactobacillus HT002	0.2442
	Oscillibacter	0.2481
	Colidextribacter	0.2529
	Parasutterella	0.2538
	Rikenellaceae_RC9_gut_group	0.2632
	Muribaculaceae	0.2813
	Lachnospiraceae_FCS020_group	0.2895
	Harryflintia	0.3252
	Bilophila	0.3345
	Clostridium GCA_900066575	0.3407
	Butyricicoccus	0.3648
	Butyricicoccus	0.3648
	Eubacterium_nodatum_group	0.4189
	Muribaculum	0.4778
	Intestinimonas	0.5251
	NK4A214_group	0.5288
	Ruminococcaceae UCG_010	0.5329
	Marvinbryantia	0.5674
	Peptococcus	0.6299
	Enterorhabdus	0.6768
	Lachnospiraceae_UCG_001	0.7733
	Incertae_Sedis	0.7764
	Ruminococcaceae UCG_005	0.8077
	Lachnispiraceae bacterium A2	0.8208
	Tuzzerella	0.8971
	Clostridia_UCG_009	0.9531

Taxa highlighted in blue can further undergo a Mann-Whitney test (see Table S5B).

Table S5B. Mann-Whitney statistical summary for microbiota data following a chronic treatment of 28 days (part A).

Level	Comparison	Name	Mann-Whitney *p*	Rank	Adjusted *q*
Family	Veh/Veh *vs* CORT/Veh	Lactobacillaceae	0.006061	1	0.04
		Prevotellaceae	0.042424	2	0.08
		Tannerellaceae	0.412121	3	0.12
		Rikenellaceae	0.648485	4	0.16
		Eubacterium_coprostanoligenes_group	>0.999999	5	0.2
	Veh/Veh *vs* CORT/Flx	Prevotellaceae	0.028283	1	0.04
		Lactobacillaceae	0.046465	2	0.08
		Tannerellaceae	0.068687	3	0.12
		Eubacterium_coprostanoligenes_group	0.418182	4	0.16
		Rikenellaceae	0.460606	5	0.2
	Veh/Veh *vs* CORT/Pruca$_{0.5}$	Lactobacillaceae	0.016162	1	0.04
		Rikenellaceae	0.214141	2	0.08
		Prevotellaceae	0.282828	3	0.12
		Eubacterium_coprostanoligenes_group	>0.999999	4	0.16
		Tannerellaceae	>0.999999	5	0.2
	Veh/Veh *vs* CORT/Pruca$_{1.5}$	Lactobacillaceae	0.006061	1	0.04
		Rikenellaceae	0.163636	2	0.08
		Eubacterium_coprostanoligenes_group	0.172727	3	0.12
		Prevotellaceae	0.527273	4	0.16
		Tannerellaceae	>0.999999	5	0.20
	CORT/Veh *vs* CORT/Flx	Rikenellaceae	0.120591	1	0.04
		Eubacterium_coprostanoligenes_group	0.2	2	0.08
		Tannerellaceae	0.22129	3	0.12
		Lactobacillaceae	0.612587	4	0.16
		Prevotellaceae	0.955089	5	0.2
	CORT/Veh *vs* CORT/Pruca$_{0.5}$	Tannerellaceae	0.040093	1	0.040
		Lactobacillaceae	0.151981	2	0.08
		Prevotellaceae	0.189277	3	0.12
		Rikenellaceae	0.396892	4	0.16
		Eubacterium_coprostanoligenes_group	>0.999999	5	0.2
	CORT/Veh *vs* CORT/Pruca$_{1.5}$	Prevotellaceae	0.037879	1	0.04
		Tannerellaceae	0.05303	2	0.08
		Eubacterium_coprostanoligenes_group	0.06993	3	0.12
		Rikenellaceae	0.072844	4	0.16
		Lactobacillaceae	>0.999999	5	0.2
	CORT/Flx *vs* CORT/Pruca$_{0.5}$	Tannerellaceae	0.004662	1	0.04
		Rikenellaceae	0.014763	2	0.08
		Lactobacillaceae	0.160528	3	0.12
		Prevotellaceae	0.160528	4	0.16
		Eubacterium_coprostanoligenes_group	0.446154	5	0.2
	CORT/Flx *vs* CORT/Pruca$_{1.5}$	Rikenellaceae	0.001243	1	0.04
		Tannerellaceae	0.002176	2	0.08
		Prevotellaceae	0.020513	3	0.12
		Eubacterium_coprostanoligenes_group	0.262471	4	0.16
		Lactobacillaceae	0.463403	5	0.2
	CORT/Pruca$_{0.5}$ *vs* CORT/Pruca$_{1.5}$	Eubacterium_coprostanoligenes_group	0.062937	1	0.04
		Lactobacillaceae	0.093862	2	0.08
		Prevotellaceae	0.53582	3	0.12
		Rikenellaceae	0.612587	4	0.16
		Tannerellaceae	0.955089	5	0.2

The largest *p* value that has $p<q$ is significant and all of the *p* values smaller than it are also significant. Highlighted in red are the taxa that maintain significance after the Benjamini-Hochberg correction. $q=(i/m)Q$ where *i* is the rank; *m* is the total number of tests; and *Q* is the false discovery rate (0.2).

Table S5B. Mann-Whitney statistical summary for microbiota data following a chronic treatment of 28 days (part B).

Level	Comparison	Name	Mann-Whitney *p*	Rank	Adjusted *q*
Genus	Veh/Veh *vs* CORT/Veh	*Ruminococcus*	0.00303	1	0.0400
		UBA1819	0.00303	2	0.0800
		Lactobacillus	0.006061	3	0.1200
		Alistipes	0.527273	4	0.1600
		Eubacterium_coprostanoligenes_group	>0.999999	5	0.2000
	Veh/Veh *vs* CORT/Flx	*UBA1819*	0.00404	1	0.0400
		Ruminococcus	0.024242	2	0.0800
		Lactobacillus	0.072727	3	0.1200
		Eubacterium_coprostanoligenes_group	0.418182	4	0.1600
		Alistipes	0.808081	5	0.2000
	Veh/Veh *vs* CORT/Puca$_{0.5}$	*Lactobacillus*	0.00404	1	0.0400
		UBA1819	0.00404	2	0.0800
		Ruminococcus	0.458586	3	0.1200
		Alistipes	0.460606	4	0.1600
		Eubacterium_coprostanoligenes_group	>0.999999	5	0.2000
	Veh/Veh *vs* CORT/Puca$_{1.5}$	*Lactobacillus*	0.006061	1	0.0400
		UBA1819	0.015152	2	0.0800
		Eubacterium_coprostanoligenes_group	0.172727	3	0.1200
		Alistipes	0.230303	4	0.1600
		Ruminococcus	0.787879	5	0.2000
	CORT/Veh *vs* CORT/Flx	*Alistipes*	0.028904	1	0.0400
		Eubacterium_coprostanoligenes_group	0.2	2	0.0800
		Ruminococcus	0.2	3	0.1200
		Lactobacillus	0.612587	4	0.1600
		UBA1819	>0.999999	5	0.2000
	CORT/Veh *vs* CORT/Puca$_{0.5}$	*Ruminococcus*	0.025641	1	0.0400
		Alistipes	0.231857	2	0.0800
		Lactobacillus	0.280963	3	0.1200
		Eubacterium_coprostanoligenes_group	>0.999999	4	0.1600
		UBA1819	>0.999999	5	0.2000
	CORT/Veh *vs* CORT/Puca$_{1.5}$	*Ruminococcus*	0.004662	1	0.0400
		Eubacterium_coprostanoligenes_group	0.06993	2	0.0800
		UBA1819	0.192308	3	0.1200
		Lactobacillus	0.534965	4	0.1600
		Alistipes	0.710373	5	0.2000
	CORT/Flx *vs* CORT/Puca$_{0.5}$	*Alistipes*	0.020668	1	0.0400
		Ruminococcus	0.110179	2	0.0800
		Eubacterium_coprostanoligenes_group	0.446154	3	0.1200
		Lactobacillus	0.573737	4	0.1600
		UBA1819	>0.999999	5	0.2000
	CORT/Flx *vs* CORT/Puca$_{1.5}$	*Alistipes*	0.005905	1	0.0400
		Ruminococcus	0.02704	2	0.0800
		Eubacterium_coprostanoligenes_group	0.262471	3	0.1200
		UBA1819	0.302564	4	0.1600
		Lactobacillus	0.778866	5	0.2000
	CORT/Puca$_{0.5}$ *vs* CORT/Puca$_{1.5}$	*Lactobacillus*	0.002176	1	0.0400
		Eubacterium_coprostanoligenes_group	0.062937	2	0.0800
		UBA1819	0.179487	3	0.1200
		Alistipes	0.53582	4	0.1600
		Ruminococcus	0.625019	5	0.2000

The largest *p* value that has $p<q$ is significant and all of the *p* values smaller than it are also significant. Highlighted in red are the taxa that maintain significance after the Benjamini-Hochberg correction. $q=(i/m)Q$ where *i* is the rank; *m* is the total number of tests; and *Q* is the false discovery rate (0.2).

Table S6. Mann-Whitney statistical summary for microbiota data following CORT treatment for 5 weeks.

Level	Name	Mann-Whitney *p*	Rank	Adjusted *q*
**Phylum**	Proteobacteria	0.1063	NA	NA
	Bacteroidota	0.3301	NA	NA
	Desulfobacterota	0.3301	NA	NA
	Firmicutes	0.3301	NA	NA
	Actinobacteriota	0.3905	NA	NA
**Family**	Clostridia_UCG_014	0.002078	1	0.01
	Ruminococcaceae	0.03357	2	0.02
	Butyricicoccaceae	0.07558	3	NA
	Ruminococcaceae_UCG_010	0.09465	4	NA
	Marinifilaceae	0.1063	5	NA
	Oscillospiraceae	0.1063	6	NA
	Rikenellaceae	0.2601	7	NA
	Sutterellaceae	0.2601	8	NA
	uncultured	0.2601	9	NA
	Desulfovibrionaceae	0.3301	10	NA
	Lactobacillaceae	0.3301	11	NA
	Eggerthellaceae	0.3905	12	NA
	Bacteroidaceae	0.414	13	NA
	Lachnospiraceae	0.414	14	NA
	Muribaculaceae	0.414	15	NA
	Prevotellaceae	0.414	16	NA
	Tannerellaceae	0.7105	17	NA
	Christensenellaceae	0.8347	18	NA
	Peptococcaceae	0.8347	19	NA
	Clostridia_vadinBB60_group	0.9399	20	NA
**Genus**	*Ruminococcus*	0.0009	1	0.0048
	UBA1819	0.0009	2	0.0097
	Clostridia_UCG_014	0.0021	3	0.0146
	Incertae_Sedis	0.0037	4	0.0195
	Intestinimonas	0.0074	5	0.0244
	Clostridia_UCG_009	0.0245	6	0.0292
	Clostridium ASF356	0.0472	7	0.0341
	Lactobacillus HT002	0.06795	8	NA
	Muribaculum	0.07552	9	NA
	Colidextribacter	0.07558	10	NA
	Ruminococcaceae_UCG_010	0.09465	11	NA
	Odoribacter	0.1063	12	NA
	Oscillibacter	0.1063	13	NA
	Clostridium GCA_900066575	0.1110	14	NA
	Lachnospiraceae bacterium A2	0.1133	15	NA
	Anaerotruncus	0.1483	16	NA
	uncultured	0.1483	17	NA
	Not_Assigned	0.1483	18	NA
	Harryflintia	0.1593	19	NA
	Alistipes	0.1986	20	NA
	Marvinbryantia	0.2511	21	NA
	Lactobacillus	0.2601	22	NA
	Parasutterella	0.2601	23	NA
	Bilophila	0.2734	24	NA
	Butyricicoccus	0.2756	25	NA
	NK4A214_group	0.3332	26	NA
	Enterorhabdus	0.3905	27	NA
	Bacteroides	0.4140	28	NA
	Muribaculaceae	0.4140	29	NA
	Ruminococcaceae UCG_005	0.4751	30	NA
	Eubacterium_xylanophilum_group	0.4816	31	NA
	Eubacterium_xylanophilum_group	0.4816	32	NA
	Alloprevotella	0.6042	33	NA
	Prevotellaceae_UCG_001	0.7105	34	NA
	Tannerellaceae	0.7105	35	NA
	Tuzzerella	0.7774	36	NA
	Ligilactobacillus	0.8077	37	NA
	Rikenellaceae_RC9_gut_group	0.8252	38	NA
	Clostridia_vadinBB60_group	0.9399	39	NA
	Lachnospiraceae_NK4A136_group	0.9399	40	NA
	Lachnospiraceae_FCS020_group	1.0000	41	NA

Comparison is Veh/Veh *vs* CORT/Veh. The largest *p* value that has $p<q$ is significant and all of the *p* values smaller than it are also significant. Highlighted in red are the taxa that maintain significance after the Benjamini-Hochberg correction. $q=(i/m)Q$ where i is the rank; m is the total number of tests; and Q is the false discovery rate (0.2).

Table S7. Mann-Whitney statistical summary for microbiota data following CORT treatment for 8 weeks.

Level	Name	Mann-Whitney *p*	Rank	Adjusted *q*
**Phylum**	Proteobacteria	0.1091	NA	NA
	Firmicutes	0.2303	NA	NA
	Bacteroidota	0.3152	NA	NA
	Desulfobacterota	0.5273	NA	NA
	Actinobacteriota	0.8885	NA	NA
**Family**	Lactobacillaceae	0.01056	1	0.0111
	Clostridia_UCG_014	0.01623	2	0.0222
	Prevotellaceae	0.04242	3	0.0333
	Bacteroidaceae	0.1091	4	NA
	Lachnospiraceae	0.1091	5	NA
	Sutterellaceae	0.1091	6	NA
	Clostridia_vadinBB60_group	0.1636	7	NA
	uncultured	0.2874	8	NA
	Muribaculaceae	0.4121	9	NA
	Tannerellaceae	0.4121	10	NA
	Desulfovibrionaceae	0.5273	11	NA
	Oscillospiraceae	0.5273	12	NA
	Peptococcaceae	0.5842	13	NA
	Butyricicoccaceae	0.6335	14	NA
	Rikenellaceae	0.6485	15	NA
	Ruminococcaceae UCG_010	0.9129	16	NA
	Ruminococcaceae	0.9273	17	NA
	Marinifilaceae	1.0000	18	NA
**Genus**	*Ruminococcus*	0.003128	1	0.0046
	UBA1819	0.003128	2	0.0093
	Lactobacillus	0.01056	3	0.0139
	Clostridia_UCG_014	0.01623	4	0.0186
	Lactobacillus HT002	0.02264	5	0.0232
	Alloprevotella	0.02939	6	0.0279
	Lachnospiraceae_NK4A136_group	0.07273	7	NA
	Harryflintia	0.1007	8	NA
	Bacteroides	0.1091	9	NA
	Parasutterella	0.1091	10	NA
	Clostridia_vadinBB60_group	0.1636	11	NA
	Eubacterium_xylanophilum_group	0.1862	12	NA
	Eubacterium_xylanophilum_group	0.1862	13	NA
	Lachnospiraceae_UCG_001	0.1862	14	NA
	Marvinbryantia	0.1862	15	NA
	Butyricicoccus	0.2086	16	NA
	Butyricicoccus	0.2086	17	NA
	NK4A214_group	0.2163	18	NA
	Rikenellaceae_RC9_gut_group	0.2303	19	NA
	Colidextribacter	0.3152	20	NA
	Prevotellaceae_UCG_001	0.3152	21	NA
	Lachnospiraceae_FCS020_group	0.3266	22	NA
	Ligilactobacillus	0.3724	23	NA
	Lachnospiraceae bacterium A2	0.4097	24	NA
	Muribaculaceae	0.4121	25	NA
	Oscillibacter	0.4121	26	NA
	Tannerellaceae	0.4121	27	NA
	Not_Assigned	0.4121	28	NA
	Incertae_Sedis	0.4436	29	NA
	Bilophila	0.4984	30	NA
	Alistipes	0.5273	31	NA
	Peptococcus	0.5842	32	NA
	Clostridium ASF356	0.6358	33	NA
	uncultured	0.6485	34	NA
	Clostridia UCG_009	0.7572	35	NA
	Ruminococcaceae UCG_005	0.7748	36	NA
	Muribaculum	0.7879	37	NA
	Ruminococcaceae UCG_010	0.9129	38	NA
	Clostridium_GCA_900066575	0.9211	39	NA
	Anaerotruncus	0.9273	40	NA
	Intestinimonas	1.0000	41	NA
	Odoribacter	1.0000	42	NA
	Tuzzerella	1.0000	43	NA

Comparison is Veh/Veh *vs* CORT/Veh. The largest *p* value that has $p<q$ is significant. and all of the *p* values smaller than it are also significant. Highlighted in red are the taxa that maintain significance after the Benjamini-Hochberg correction. $q=(i/m)Q$ where *i* is the rank; *m* is the total number of tests; and *Q* is the false discovery rate (0.2).

III. SUPPLEMENTAL FIGURES

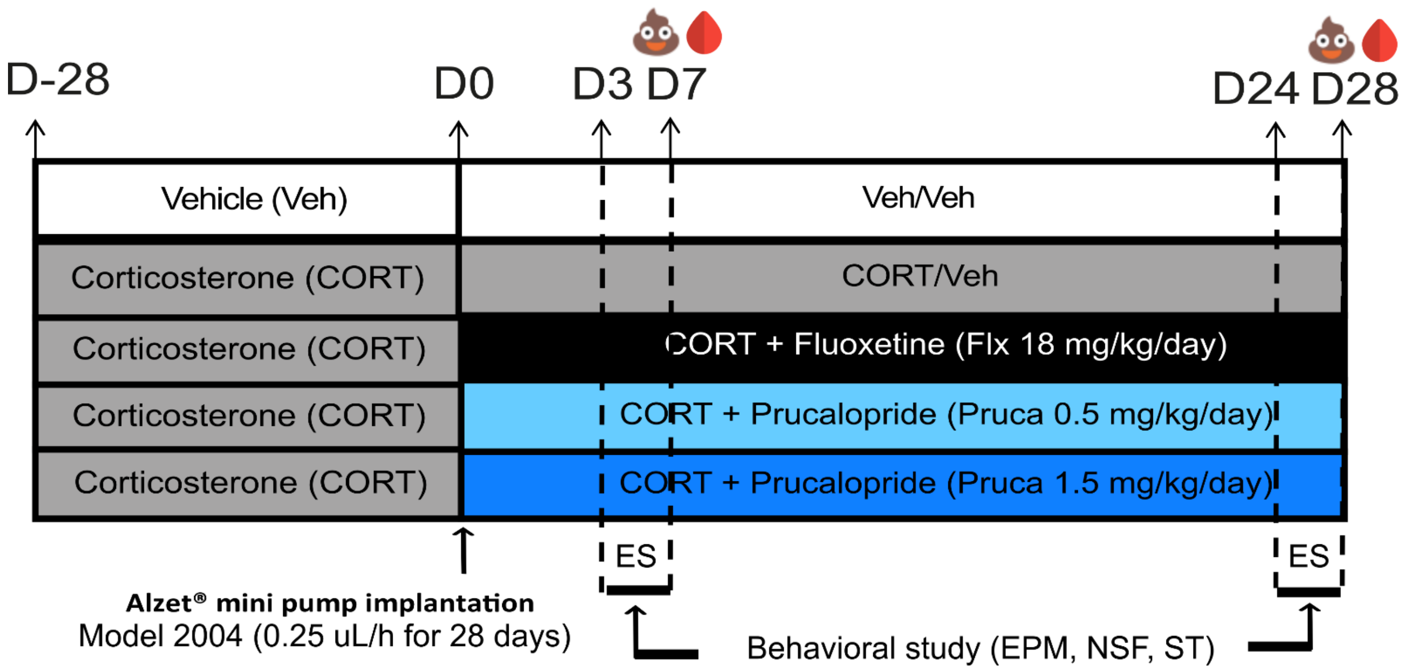

Figure S1. Experimental timeline.

In place of normal drinking water, grouped-housed male C57BL/6JRj mice were presented with vehicle (Veh, 0.45% hydroxypropyl-β-cyclodextrin) or corticosterone (CORT, 35 µg/ml, equivalent to about 5 mg/kg/day) for 56 days in the presence or absence of a 5-HT_4 agonist, prucalopride ($Pruca_{0.5}$ and $Pruca_{1.5}$ mg/kg/day, Alzet minipump subcutaneous, 2004 model) or fluoxetine (Flx), 18 mg/kg/day for the last for four weeks of the protocol (28 days). We investigated whether the behavioral changes induced by chronic CORT were reversed after subchronic (days 3 to 7) or chronic (days 24 to 28) drug treatment. The same animal was successively tested in three different behavioral paradigms, from the least to the most aversive [the Elevated Plus Maze (EPM, predictive of an anxiolytic like-activity) at day 3 and day 24, the Novelty Suppressed Feeding (NSF, predictive of a mixte anxiolytic/antidepressant like-activity) at day 5 and day 26 and the Splash test (ST, predictive of an anxiolytic like-activity) at day 6 and day 27. Emotional Z-scores (EPM, NSF and ST) were then calculated by averaging individual Z-scores and averaged to obtain the emotionality score (ES) after subchronic and chronic treatments. At the end of each behavioral session on day 7 and day 28, plasma and faeces were collected for drug and corticosterone levels and for 16S sequencing analysis in the morning (from 7am to 12pm).

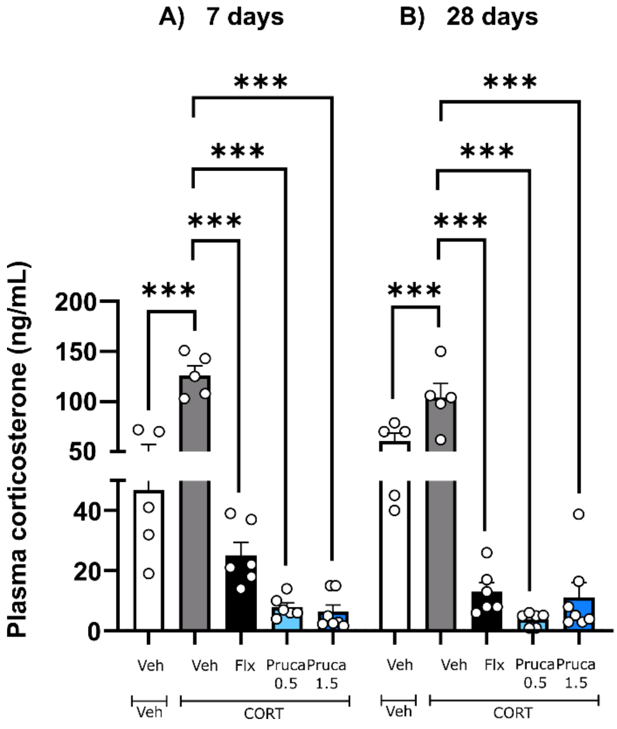

Group	Subchronic (7 days)		Chronic (28 days)	
	Mean (ng/mL)	SEM (n)	Mean (ng/mL)	SEM (n)
Veh/Veh	35.70	9.14 (5)	60.80	6.88 (5)
CORT/Veh	126.00	8.41 (5)	104.00	12.52 (5)
CORT/Flx	25.17	3.85 (6)	13.00	2.81 (6)
CORT/$Pruca_{0.5}$	7.83	1.47 (6)	3.88	0.85 (6)
CORT/$Pruca_{1.5}$	6.34	2.10 (7)	11.20	4.56 (7)

Figure S2. Effect of a subchronic and chronic prucalopride treatment on plasma corticosterone levels.

(A) Plasma corticosterone (CORT) was measured after the first behavioral session (days 7) using liquid chromatography coupled to tandem mass spectrometry (LC-MS/MS). One-way ANOVA $F_{(4,24)}=65.95$ $p<0.0001$. **(B)** Plasma corticosterone (CORT) was measured after the second behavioral session (days 28) using liquid chromatography coupled to tandem mass spectrometry (LC-MS/MS). One-way ANOVA $F_{(4,24)}=37.15$ $p<0.0001$. Multiple comparisons are performed via Fisher's LSD test: *$p<0.05$; **$p<0.01$; ***$p<0.001$. Data are expressed as mean + SEM (Veh/Veh n=5, CORT/Veh n=5, CORT/Flx n=6, CORT/$Pruca_{0.5}$ n=6, CORT/$Pruca_{1.5}$ n=7).

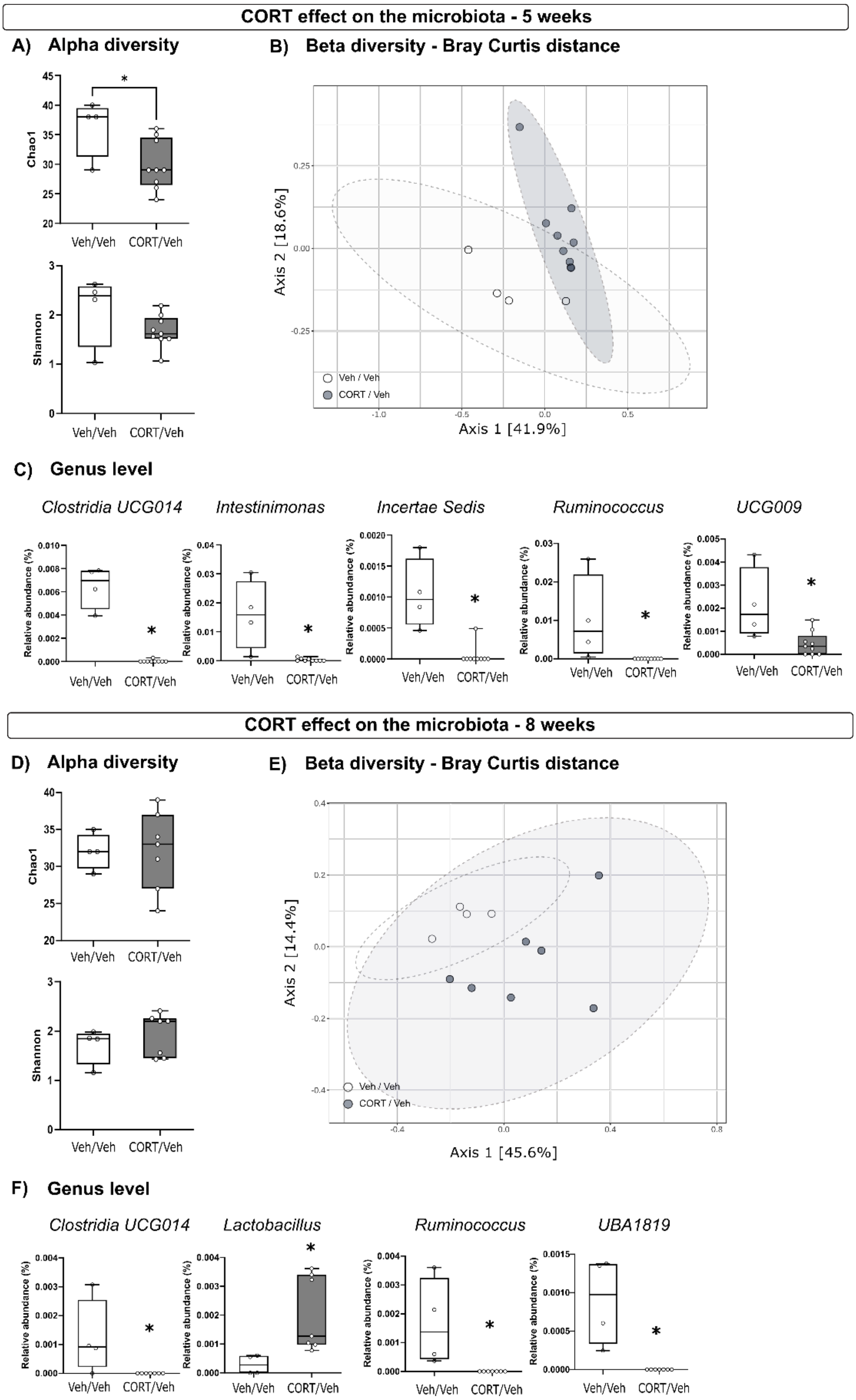

Figure S3. Chronic corticosterone administration alters the gut microbiota composition.

(A-C) Gut microbiota composition is altered after a 5-week corticosterone (CORT) treatment. **(A)** Alpha diversity: Mann-Whitney test for Chao1 U=4.5 *p*=0.034. **(B)** Beta diversity, principal coordinate analysis of Bray Curtis compiled distance matrix of all genera: PERMANOVA $F_{(1,12)}$=4.03 *p*=0.003. **(C)** Microbiota changes at genus level. **p*<0.05 *vs* Veh/Veh.

(D-F) Gut microbiota composition is altered after an 8-week corticosterone (CORT) treatment. **(D)** Alpha diversity: Mann-Whitney test for Chao1 and Shannon not significant. **(E)** Beta diversity, principal coordinate analysis of Bray Curtis compiled distance matrix of all genera: PERMANOVA $F_{(1,12)}$=2.88 *p*=0.02. **(F)** Microbiota changes at genus level. *p<0.05 *vs* Veh/Veh. Data are expressed as median ± min-to-max values (Veh/Veh n=4, CORT/Veh n=9). Data are analyzed using Mann-Whitney non-parametric U-test and corrected for multiple comparisons using the Benjamin-Hochberg false discovery rate (FDR) method.

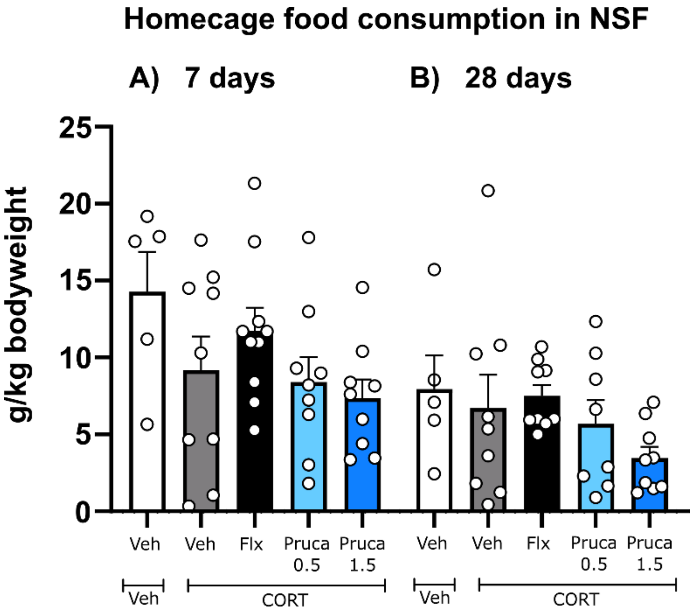

Figure S4. Homecage food consumption related to the NSF test.

(A) Homecage food consumption was measured right after the NSF test in subchronically treated mice. One-way ANOVA revealed no significant differences $F_{(4,37)}$=2.01 *p*=0.112. **(B)** Homecage food consumption was measured right after the NSF test in chronically treated mice. One-way ANOVA revealed no significant differences $F_{(4,35)}$=1.39 *p*=0.256. Data are expressed as mean + SEM (Veh/Veh n=5, CORT/Veh n=9, CORT/Flx n=10, CORT/$Pruca_{0.5}$ n=8-9, CORT/$Pruca_{1.5}$ n=9).

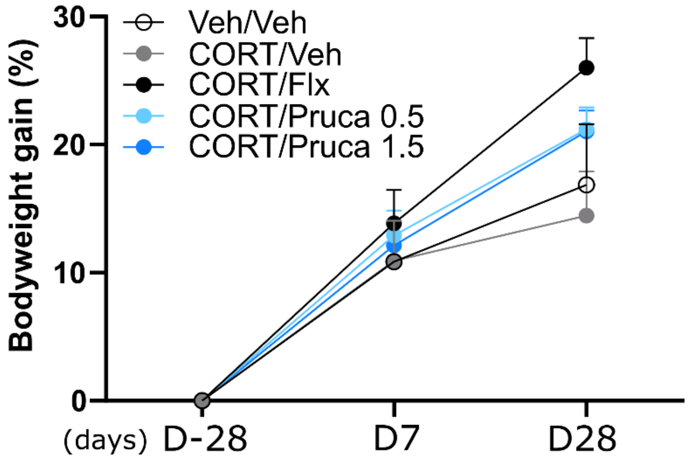

Figure S5. Changes in bodyweight gain across the experimental timeline.

Bodyweight gain was calculated based on the baseline measure (D-28). Repeated-measures One-way ANOVA revealed no significant differences due to treatments $F_{(1.01,2.03)}$=2.03 *p*=0.289. Data are expressed as mean + SEM (Veh/Veh n=5, CORT/Veh n=9, CORT/Flx n=10, CORT/$Pruca_{0.5}$ n=9, CORT/$Pruca_{1.5}$ n=9).
